# An integrated genomics analysis of epigenetic subtypes in human breast tumors links DNA methylation patterns to chromatin states in normal mammary cells

**DOI:** 10.1186/s13058-016-0685-5

**Published:** 2016-02-29

**Authors:** Karolina Holm, Johan Staaf, Martin Lauss, Mattias Aine, David Lindgren, Pär-Ola Bendahl, Johan Vallon-Christersson, Rosa Bjork Barkardottir, Mattias Höglund, Åke Borg, Göran Jönsson, Markus Ringnér

**Affiliations:** Division of Oncology and Pathology, Department of Clinical Sciences Lund, Lund University, Lund, Sweden; CREATE Health Centre for Translational Cancer Research, Lund University, Lund, Sweden; Division of Translational Cancer Research, Department of Laboratory Medicine Lund, Lund University, Lund, Sweden; Department of Pathology, Landspitali-University Hospital, Reykjavik, Iceland

**Keywords:** Breast cancer, DNA methylation, Histone modification, Gene expression, Copy number alteration, Mutation, *BRCA1*, *BRCA2*, ENCODE, The Cancer Genome Atlas

## Abstract

**Background:**

Aberrant DNA methylation is frequently observed in breast cancer. However, the relationship between methylation patterns and the heterogeneity of breast cancer has not been comprehensively characterized.

**Methods:**

Whole-genome DNA methylation analysis using Illumina Infinium HumanMethylation450 BeadChip arrays was performed on 188 human breast tumors. Unsupervised bootstrap consensus clustering was performed to identify DNA methylation epigenetic subgroups (epitypes). The Cancer Genome Atlas data, including methylation profiles of 669 human breast tumors, was used for validation. The identified epitypes were characterized by integration with publicly available genome-wide data, including gene expression levels, DNA copy numbers, whole-exome sequencing data, and chromatin states.

**Results:**

We identified seven breast cancer epitypes. One epitype was distinctly associated with basal-like tumors and with *BRCA1* mutations, one epitype contained a subset of *ERBB2*-amplified tumors characterized by multiple additional amplifications and the most complex genomes, and one epitype displayed a methylation profile similar to normal epithelial cells. Luminal tumors were stratified into the remaining four epitypes, with differences in promoter hypermethylation, global hypomethylation, proliferative rates, and genomic instability. Specific hyper- and hypomethylation across the basal-like epitype was rare. However, we observed that the candidate genomic instability drivers *BRCA1* and *HORMAD1* displayed aberrant methylation linked to gene expression levels in some basal-like tumors. Hypomethylation in luminal tumors was associated with DNA repeats and subtelomeric regions. We observed two dominant patterns of aberrant methylation in breast cancer. One pattern, constitutively methylated in both basal-like and luminal breast cancer, was linked to genes with promoters in a Polycomb-repressed state in normal epithelial cells and displayed no correlation with gene expression levels. The second pattern correlated with gene expression levels and was associated with methylation in luminal tumors and genes with active promoters in normal epithelial cells.

**Conclusions:**

Our results suggest that hypermethylation patterns across basal-like breast cancer may have limited influence on tumor progression and instead reflect the repressed chromatin state of the tissue of origin. On the contrary, hypermethylation patterns specific to luminal breast cancer influence gene expression, may contribute to tumor progression, and may present an actionable epigenetic alteration in a subset of luminal breast cancers.

**Electronic supplementary material:**

The online version of this article (doi:10.1186/s13058-016-0685-5) contains supplementary material, which is available to authorized users.

## Background

Breast cancer is the most common cancer and one of the leading causes of cancer death among women. The disease is heterogeneous, both clinically and molecularly. A large number of molecular studies have characterized breast cancer on the basis of data derived from one or two genome-wide measurement platforms, typically using gene expression or DNA copy number platforms [1, 2]. Arguably the most influential finding to emerge from these studies is the robust identification of five gene expression–based molecular subtypes of breast cancer: two estrogen receptor (ER)-positive subtypes separated mainly by relatively low (luminal A) and high (luminal B) expression of proliferation-related genes, a subtype enriched for *ERBB2*-amplified tumors [human epidermal growth factor receptor 2 (HER2)-enriched], a subtype associated with triple-negative [lacking expression of ER, progesterone receptor (PR), and HER2] tumors (basal-like), and a subtype with an expression profile similar to that of normal breast tissue (normal-like) [3]. Later studies using multiple different platforms, including exome sequencing, DNA copy number arrays, DNA methylation arrays, and gene expression arrays, have highlighted the importance of integrating information across platforms to identify key characteristics of the molecular subtypes of breast cancer [4].

DNA methylation patterns and chromatin states are epigenetic features often found to be altered in cancer cells [5]. The breast cancer molecular subtypes have been found to be associated with characteristic DNA methylation patterns on the basis of limited panels of CpG sites [6–8]. Typically, three major DNA methylation subtypes of breast tumors have been identified. One group is characterized by the lowest levels of DNA methylation and is associated with basal-like tumors. A second group is characterized by hypermethylation of promoter CpG sites and is associated with luminal B tumors. A third group is associated with luminal A tumors, whereas the HER2-enriched and normal-like gene expression–based subtypes have been found to have limited association with DNA methylation subtypes. Later, these observations were confirmed using genome-wide sets of CpG sites located primarily in promoter regions [4] as well as across the entire genome [9].

There are many links between chromatin states and DNA methylation [5]. In cancer, widespread correlated changes in DNA methylation patterns and chromatin states have been observed [10]. As these features collectively are associated with whether genes are transcriptionally active or inactive, they may underlie phenotypic changes observed in cancer cells. Furthermore, recent sequencing efforts have identified mutations of genes leading to altered epigenetic patterns for many tumor types [10]. In breast cancer specifically, a number of links between DNA methylation and chromatin state have been observed. For example, promoters that are hypermethylated are often in lineage-commitment genes that in embryonic stem cells are in a transcription-ready bivalent chromatin state characterized by both active and repressive marks [11, 12]. Another example is the observation of extensive chromatin state changes upon loss of DNA methylation in breast cancer coupled with maintaining these hypomethylated regions as transcriptionally silent [13].

However, less is known about how DNA methylation patterns and epigenetic states on a genome-wide scale are coupled with breast cancer heterogeneity as reflected in the breast cancer subtypes. The development of platforms for genome-wide characterization of cells at many levels, together with large public datasets of normal and malignant breast samples, have provided opportunities to address this question. In the present study, we investigated breast cancer heterogeneity on the basis of genome-wide DNA methylation profiles of human tumors and integrated our findings with various types of molecular data, including chromatin states in both embryonic stem cells and human mammary epithelial cells (HMECs) generated in the ENCODE project [14]. In a discovery cohort with DNA methylation profiles from 188 samples, we identified seven epitypes of breast cancer that were validated in 669 independent samples from The Cancer Genome Atlas (TCGA) breast cancer project [4]. By integrating analyses across multiple platforms, we show that the epitypes are associated with specific gene expression subtypes, mutations, and DNA copy number aberrations (CNAs). To characterize epitype-specific hyper- and hypomethylation patterns, we identified sets of CpG sites that display differential methylation status between normal breast tissue and tumors of an epitype. These analyses revealed that DNA hypermethylation in luminal and basal-like tumors occurs in different chromatin contexts with different underlying regulatory potential in stem and mammary epithelial cells. Moreover, hypomethylation in luminal tumors was associated with DNA repeats and subtelomeric regions. Our results highlight links between breast cancer subtypes and the epigenome that could improve understanding of biological mechanisms underlying breast cancer heterogeneity and could eventually contribute to diagnostics and therapeutic interventions.

## Methods

### Sample material for methylation analysis

Fresh frozen breast tumor tissues (*n* = 188) obtained from the Southern Sweden Breast Cancer Group tissue bank at the Department of Oncology and Pathology, Skåne University Hospital (Lund, Sweden), and from the Department of Pathology, Landspitali University Hospital (Reykjavik, Iceland), were used as a discovery cohort. The 188 breast tumor tissues were from 181 unique female patients (183 primary tumor samples, 2 metastatic samples, and 3 locoregional recurrences; for 3 patients a primary and a recurrent sample were included, and for 4 patients 2 different primary tumors were included). The study was approved by the regional ethics committee in Lund, which waived the requirement for informed consent for the study (numbers LU240-01 and 2009/658), as well as by the Icelandic Data Protection Committee and the National Bioethics Committee of Iceland. For Icelandic patients, written informed consent was obtained according to Icelandic national guidelines.

Breast invasive carcinomas from the TCGA project with 450K methylation data available (based on TCGA update 27 September 2013) were used as a validation cohort [4]. Replicated tumors were removed and female patients selected, resulting in a validation cohort consisting of 669 breast carcinomas from 666 unique female patients (665 primary tumor samples and 4 metastatic samples; for 3 patients a primary and a metastatic sample were included). For the normal cohort, 96 normal specimens originating from normal breast tissue from 96 different female patients from the TCGA project were used (90 of these patients also have a tumor sample in the validation cohort).

DNA from human mammary fibroblasts, HMECs, human mammary endothelial cells (ScienCell Research Laboratories, Carlsbad, CA, USA), and peripheral blood leukocytes (Promega, Madison, WI, USA) was used to generate a cohort of normal cell types. DNA methylation data from subpopulations of human blood cells generated by Reinius et al. [15] were downloaded from the National Center for Biotechnology Information (NCBI) Gene Expression Omnibus (GEO) [16] accession number [GEO:GSE35069].

### DNA methylation analysis

Genome-wide methylation data for the discovery cohort and the cohort of normal cell types were generated at SCIBLU Genomics, Lund University, using the Illumina Infinium HumanMethylation450 BeadChip Array (Illumina, San Diego, CA, USA) according to the manufacturer’s instructions. For the discovery cohort, DNA was extracted as previously described [6]. DNA was treated with bisulfite using the EZ DNA Methylation Kit (Zymo Research, Irvine, CA, USA) according to the manufacturer’s instructions. DNA methylation data for the discovery cohort and the cohort of normal cell types are available in the NCBI GEO [16] under accession numbers [GEO:GSE75067] and [GEO:GSE74877], respectively.

The 450K methylation data were processed similarly for all cohorts. Methylated and unmethylated signal intensities were obtained from GenomeStudio (Illumina) for the discovery and normal cell type cohorts, and from TCGA methylation level 2 data for the validation and normal breast tissue cohorts. Signal intensities were converted into β values [β = methylated/(methylated + unmethylated)] representing the methylation levels. CpG sites with detection *p* values greater than 0.05 or the number of beads for a channel fewer than 3 were considered missing measurements, and β values were set to “NA” (with the exception that the number of beads was not available for the TCGA cohorts). No sample had more than 10,000 missing values (discovery cohort range 835–9438, validation cohort range 214–4746, normal breast tissue cohort range 258–2700, normal cell type cohort range 758–1278). For the blood subpopulation data, β values were obtained as processed in the NCBI GEO.

Adjustment for bias between Infinium I and II assay CpG probes was performed by using a peak normalization algorithm. Briefly, for each sample, we performed a peak-based correction of Illumina I and II chemical assays inspired by Dedeurwaerder et al. [17] as previously described [18]. For each chemical assay separately, we smoothed the β values (Epanechnikov smoothing kernel) to estimate unmethylated and methylated peaks. The unmethylated peak was moved to 0 and the methylated peak to 1 using linear scaling, with β values in between stretched accordingly. β values less than 0 were set to 0 and values greater than 1 were set to 1.

A DNA hypermethylation score was calculated as described elsewhere [19]. The hypermethylation score was calculated for two sets of CpG sites: a global score in which all CpG sites on the platform contributed, and a promoter CpG island score in which CpG sites with both Illumina annotation TSS1500 or TS200 and Illumina CpG island annotation contributed.

### Identification and validation of breast cancer epitypes

Unsupervised bootstrap consensus clustering was performed to identify DNA methylation subgroups of tumors using 2000 bootstrap iterations as described elsewhere [20]. The ward.D agglomerative method with Pearson correlation–based distance in the R package hclust was used for both the inner clustering (based on methylation patterns) and the outer clustering (based on bootstrap coclustering frequencies). DNA methylation centroids for an epitype were constructed by taking the average β value for each CpG site across the tumors in the epitype in the discovery cohort. Pearson correlations between tumors in the validation cohort and the centroids were calculated. Each tumor in the validation cohort was classified into an epitype on the basis of the centroid to which the correlation was largest. Principal component analysis was used to determine that no technical artifacts influenced the methylation data or the epitypes and that the epitypes were associated with the dominant variation in genome-wide methylation data [21].

### Gene expression data analysis

Gene expression data from oligonucleotide microarrays were available for 158 of the tumors in the discovery cohort as part of accession number [GEO:GSE25307], which encompasses 577 breast tumors [22]. The normalized gene expression values (mean-centered across 577 tumors) in accession number [GEO:GSE25307] were used. Probes were mapped to Entrez Gene IDs, and the probe with the largest variation in expression across the 577 tumors was selected for each gene, resulting in relative gene expression levels for 7499 genes in the discovery cohort. TCGA RNAseq v2 level 3 data were available for a total of 994 tumors and 106 normal breast tissue samples, including 661 of the 669 tumors in the validation cohort. The gene-normalized RSEM count estimates were offset by a pseudocount of 1, log_2_-transformed, and mean-centered across the 994 tumor samples to generate relative gene expression levels for 20,531 genes in the validation cohort. For some analyses, we were interested in comparing estimates of the expression levels of different genes and therefore could not use relative expression levels across tumors. In these analyses, we took the effective transcript length into account by using the gene RSEM scaled estimates (tau) from the TCGA data transformed into transcripts per million (TPM), and used log_2_(TPM + 1) as a measure of gene expression [23]. The R package genefu was used to assign expression-based molecular subtype to tumor samples on the basis of PAM50 using relative expression levels in both the discovery and validation cohorts [24]. Expression data for 35 and 50 of the 50 PAM50 genes were available in the discovery and validation cohorts, respectively. The R package iC10 was used to assign IntClust groups to tumor samples in both the discovery and validation cohorts [25]. For each cohort, the iC10 package was run with the following settings: expression data only, probe mapping based on gene symbols, and normalizing each probe to a Z-score. Expression data for 346 and 584 of the 612 iC10 genes were available in the discovery and validation cohorts, respectively. The activity of eight gene modules, representing transcriptional programs in breast cancer, was calculated in each tumor in both the discovery and validation cohorts as the average relative expression level of the genes in a module [26]. Genes in modules were mapped to genes in expression data based on Entrez Gene ID.

### Correlation between DNA methylation and gene expression

We calculated correlations between methylation and relative gene expression levels using the validation cohort because the number of genes was limited on the expression platform used in the discovery cohort. Matching on gene symbol, 324,991 CpG sites were associated with a unique gene in the TCGA gene expression data and displayed variation in methylation levels across the validation cohort. Pearson correlations of 0.2 and −0.2 between gene expression and methylation levels were associated with *p* values much less than 10^−6^. Hence, correcting for multiple hypothesis testing, less than 1 CpG site having a Pearson correlation greater than 0.2 or less than −0.2 is expected by chance across the 661 tumors.

### Functional classification of gene sets

Enrichment of functional classification of genes in identified gene sets was analyzed using the DAVID Functional Annotation Tool [27] with the default Homo sapiens background and the false discovery rate (FDR) option to correct for multiple hypothesis testing. Gene set enrichment analysis was used to investigate the overlap of genes in identified gene sets with genes in 10,348 gene sets collected in the Molecular Signatures Database (MSigDB) [28]. In these analyses, CpG annotation data obtained from Illumina were used to map CpG sites to genes, and only CpG sites mapping to a unique gene were included.

### Processing of human genome data

Chromatin states in human embryonic stem cells (H1hESCs) and HMECs, as well as peak calls for DNase I hypersensitive sites and EZH2 binding sites in HMECs from the uniform pipeline, all generated by the ENCODE consortium, were obtained using the UCSC Genome Browser [14, 29]. CpG sites were mapped to chromosome regions with information from ENCODE using the R package GenomicRanges [30]. CpG sites were mapped to DNA repeat regions using the repeats_rmsk_hg19.txt table in the UCSC Genome Browser.

### *BRCA1* and *HORMAD1* promoter methylation analysis

*BRCA1* promoter methylation analysis was performed using the 450K methylation data. To identify informative CpG sites, we screened all 44 CpG sites on the platform located within *BRCA1* transcripts or 1 kb upstream for negative correlation (Pearson correlation less than −0.2) with *BRCA1* gene expression levels using the validation cohort. We identified 21 informative CpG sites. All informative CpG sites were located within 1 kb centered on the *BRCA1* transcription start site. Tumors were classified as *BRCA1* promoter methylated if the average β value for the informative CpG sites was greater than 0.2. The average β value for the informative CpG sites ranged from 0.004 to 0.03 across the 96 normal tissue samples in the normal cohort. To validate BRCA1 promoter status, we used data available for 71 tumors from a previous study in the discovery cohort and obtained with a PSQ HS 96 pyrosequencing system (Biotage, Uppsala, Sweden) as described [22]. *HORMAD1* promoter methylation analysis was performed in the same way as it was for *BRCA1*. We identified seven *HORMAD1* informative CpG sites among nine CpG sites located within *HORMAD1* transcripts or 1 kb upstream. All informative CpG sites were located within 1 kb centered on the *HORMAD1* transcription start site. Tumors were classified as *HORMAD1* promoter unmethylated if the average β value for the informative CpG sites was less than 0.8. The average β value for the informative CpG sites ranged from 0.91 to 0.99 across the 96 normal tissue samples in the normal cohort.

### Somatic mutation analysis

Somatic mutations from exome sequencing were available from TCGA for 645 of the 669 tumors in the validation cohort [mutation annotation format (MAF) file, curated level 2 data, version 2.1.1.0]. For some tumors, the MAF file contained mutations called from multiple exome sequencing experiments with different reference samples or different tumor aliquots. (For 34 of the tumors mutations were from 2 experiments, and for 1 tumor mutations were from 3 experiments.) We called gene mutations when genes were mutated in at least one experiment for the tumor, and the total number of single-base substitutions for each tumor was calculated as the average for the multiple experiments.

### Copy number analysis

Copy number estimates and CNAs obtained from bacterial artificial chromosome (BAC) arrays were available for 180 of 188 tumors in the discovery cohort from previous studies [22, 31, 32]. Affymetrix Genome-Wide Human SNP Array 6.0 (Affymetrix, Santa Clara, CA, USA) level 3 data were available from TCGA for 660 of 669 tumors in the validation cohort and were used to generate copy number estimates and CNAs as described elsewhere [33]. The fraction of the genome altered (FGA) by copy number alterations was estimated as the number of probes with copy number gain or loss divided by the total number of probes for the platform. Amplifications were identified using a previously defined set of significant DNA CNAs in breast cancer [31]. This set was identified using GISTIC [34]. GISTIC regions with an average copy number estimate of probes in the region greater than 0.8 were called as amplifications in both the discovery and validation cohorts. Complex arm-wise aberration index (CAAI) scores were calculated for each tumor as described by Russnes et al. [35]. A case was classified as CAAI-positive if one or more chromosome arms were affected by complex alterations with a CAAI score greater than 2 for samples in the discovery cohort or greater than 4 in the TCGA cohort. The reason for the difference in cutoff between the cohorts is due to the different platforms from which the copy number data were generated (Affymetrix Genome-Wide Human SNP Array 6.0 for TCGA, BAC arrays for the discovery cohort). The different platforms have different responses (platform-related characteristics) to copy number change (amplitude), and this amplitude is an important variable in the CAAI calculation.

### Statistical analysis

Wilcoxon tests, Kruskal-Wallis tests, χ^2^ tests, *t* tests, and Fisher’s exact tests were performed in R. Adjustment of *p* values for multiple-testing correction of these statistical tests was performed using p.adjust in R with the Benjamini-Hochberg method to control the FDR [36]. Survival analysis was performed in R using the survival package. Survival functions for patients stratified by epitypes were estimated using the Kaplan-Meier estimator and compared using the log-rank test. In the survival analysis, 169 samples (first primary tumor with available survival data) were included for the discovery cohort and 654 samples (primary tumor with available survival data) were included for the validation cohort.

## Results

### Identification of CpG sites with breast cancer–specific methylation patterns

We compared the DNA methylation status of more than 480,000 CpG sites between 188 breast cancer samples (Table 1, discovery cohort) and 96 normal breast tissue specimens (normal cohort). To identify CpG sites with different methylation levels in tumors as compared with normal samples, we first identified 284,627 CpG sites as being either methylated (β > 0.7) or unmethylated (β < 0.3) across all 96 samples in the normal cohort (allowing for 2 missing values). Among these CpG sites, we identified 2108 CpG sites that changed methylation status in at least 5 % (*n* = 10) of the breast tumors (Additional file 1: Table S1). Of these, 1016 CpG sites were methylated in breast cancer (β < 0.3 in the normal samples and β > 0.7 in the tumor samples) and 1092 CpG sites were unmethylated in breast cancer (β > 0.7 in the normal samples and β < 0.3 in the tumor samples). We observed that more than 95 % of these 2108 CpG sites also changed methylation status in at least 5 % of the tumors in the TCGA validation cohort. Because we identified CpG sites that display tumor-specific methylation robustly across cohorts from different populations, including the TCGA validation cohort with matched tumor-normal pairs, we concluded that the influence of single-nucleotide polymorphisms and other germline variants is limited on the identified CpG sites.

**Table 1:** Patient and tumor characteristics of included cohorts

Characteristic	Discovery cohort (*n*)	Validation cohort (*n*)
Total number of samples	188	669
Unique patients	181	666
Primary tumors	183	665
Recurrent tumors	5	4
Median age, years	48	58
Estrogen receptor status		
Positive	97	474
Negative	77	142
Tumor size		
T1	66	176
T2	81	371
T3	5	86
Node status		
Positive	59	356
Negative	91	290
Histological type		
Ductal	120	446
Lobular	8	134
Medullary	3	5
Mixed	9	24
Other	18	44
Molecular subtype		
Luminal A	41	251
Luminal B	30	144
HER2-enriched	32	73
Basal-like	44	126
Normal-like	11	67

*HER2* human epidermal growth factor receptor 2

### Unsupervised identification of seven epitypes in breast cancer

**Fig. 1:**
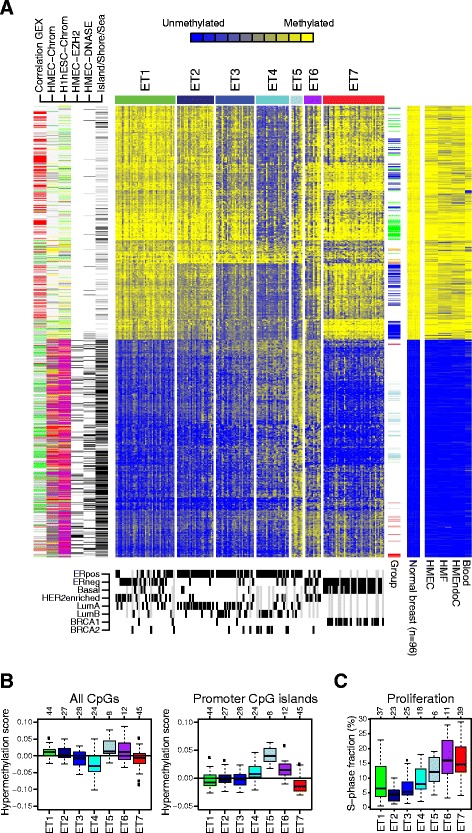
Identification of seven DNA methylation epitypes in breast cancer. **a** DNA methylation epitypes in the discovery cohort based on bootstrap clustering of 2108 CpG sites with breast cancer–specific methylation levels. The heat map displays β values (rows) ranging from unmethylated (*blue*) to methylated (*yellow*) for three sample groups (columns) comprising 188 breast tumors divided into 7 epitypes by bootstrap clustering, 96 normal breast tissues from The Cancer Genome Atlas, and 4 normal cell types (*HMEC* human mammary epithelial cells, *HMF* human mammary fibroblasts, *HMEndoC* human mammary endothelial cells, *Blood* blood leukocytes). Sample annotations at the bottom display estrogen receptor status, gene expression subtypes, germline mutations in *BRCA1* and *BRCA2* (*black* = yes, *white* = no, *gray* = NA). CpG tracks on the left side: *GEX* correlation between DNA methylation and gene expression levels across the validation cohort (*red* = positive correlation, *green* = negative correlation, *gray* = low correlation, *white* = no associated gene); *HMEC-Chrom* and *H1hESC-Chrom* chromatin states in human mammary epithelial cells and H1 human embryonic stem cells, respectively (*red* = active promoter, *purple* = poised promoter, *gray* = Polycomb-repressed, *yellow* = enhancer, *green* = transcribed, *blue* = insulator, *white* = heterochromatin); *HMEC-EZH2* EZH2 targets in human mammary epithelial cells; *HMEC-DNASE* accessible DNA in human mammary epithelial cells (*black* = yes, *white* = no); CpG island track (*black* = island, *gray* = shore/shelf, *white* = open sea). CpG track on the right side: *Group* CpG sites with epitype-specific methylation patterns (*red* = methylated in ET7, *light blue* = methylated in ET5, *green* = demethylated in ET4, *blue* = demethylated in luminal epitypes, *orange* = demethylated in ET7). **b** Global hypermethylation scores for all CpG sites (*left*) and all CpG sites in promoters and CpG islands (*right*) across the epitypes. **c** Proliferative rates of tumors across the epitypes. In (**b**) and (**c**), the number of tumors in each epitype is shown at the top. *ER* estrogen receptor, *HER2* human epidermal growth factor receptor 2

We performed unsupervised bootstrap consensus clustering analysis based on the 2108 CpG sites with tumor-specific methylation levels, and we identified 7 clusters (hereafter referred to as epitypes ET1–ET7) of breast cancer samples in the discovery cohort (Fig. 1a, Additional file 2: Figure S1A). In addition to basing the 7 epitypes on 2000 bootstrap iterations of clustering, we tested the robustness of the epitypes in several ways. First, we evaluated the number of epitypes by performing bootstrap clustering analysis looking for three to ten clusters. Typically, unstable clusters, clusters that clearly contained subclusters, or clusters with fewer than five tumors were identified. However, for three and seven clusters, robust solutions were obtained. We decided upon the solution with the largest number of robust clusters (seven clusters), which provided a relatively consistent subdivision from the three-cluster solution (Additional file 2: Figure S1B). Second, the seven epitypes were robust across different CpG sets in unsupervised bootstrap clustering analysis (Additional file 2: Figure S1C, D). Furthermore, although the epitypes were identified using tumor-specific CpG sites, they were associated with the dominant variation in the genome-wide DNA methylation levels as measured by the entire platform (Additional file 2: Figure S1E). Third, technical factors, such as bisulfite conversion plate or BeadChip array, influenced neither the genome-wide methylation data nor the epitypes (Additional file 2: Figure S1E–G). ET1 showed a methylation pattern most similar to normal samples, ET4 a global hypomethylation pattern, ET5 a promoter CpG island hypermethylation pattern, and ET7 a promoter CpG island hypomethylation pattern (Fig. 1b). The proliferative rates of the tumors, as measured by the fraction of cells in S phase determined by flow cytometry, increased from ET2 to ET7 (Fig. 1c).

### Validation of breast cancer epitypes

We constructed a classifier for the 7 epitypes using the discovery cohort and the 2108 tumor-specific CpG sites. Next, we classified 669 independent breast tumors in the TCGA validation cohort (Table 1). ET4 showed a global hypomethylation pattern, ET5 a promoter CpG island hypermethylation pattern, and ET7 a promoter CpG island hypomethylation pattern also in the validation cohort (Additional file 2: Figure S2A). The epitype classification explained the dominant variation in the genome-wide DNA methylation levels (Additional file 2: Figure S2B). Notably, the epitypes contributed more to the total variation in DNA methylation than clinicopathological and technical factors. Again, the epitypes were not associated with technical factors such as TCGA batch and BeadChip array (Additional file 2: Figure S2C-D). Moreover, we performed unsupervised bootstrap clustering analysis to independently derive epitypes in the validation cohort, following the same approach as in the discovery cohort. This analysis resulted in eight clusters of tumors that overlapped extensively with the classification into the seven epitypes (Additional file 2: Figure S2E). The main difference was that in the larger validation cohort there was support to robustly split ET3 into two groups. Together, these results demonstrate that the breast cancer epitypes are reproducible and can be robustly identified across independent cohorts.

### Epitypes are associated with gene expression phenotypes

**Fig. 2:**
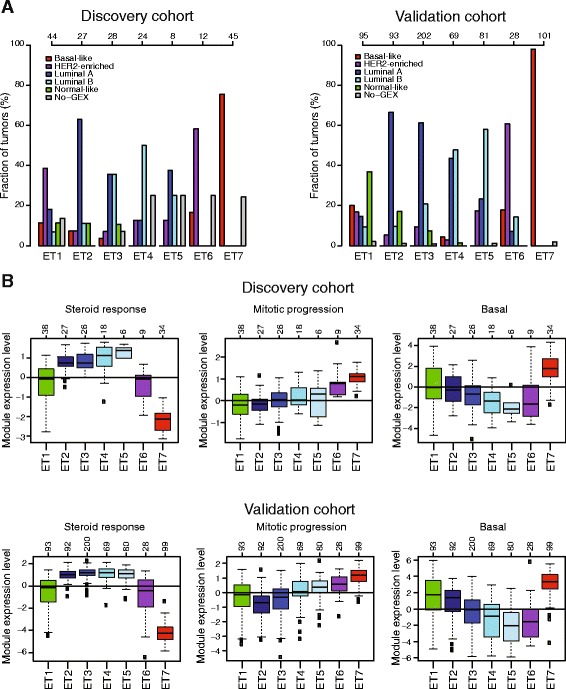
Gene expression phenotypes of breast cancer epitypes. **a** Percentage of tumors in an epitype classified into each expression-based molecular subtype. *No-GEX* samples without available gene expression data. **b** Expression levels of three gene modules stratified by epitypes. In all panels, the discovery and validation cohorts are shown separately and numbers of analyzed tumors in each epitype are shown at the top. *HER2* human epidermal growth factor receptor 2

The seven epitypes were associated with the molecular subtypes of breast cancer in both the discovery and validation cohorts (Fig. 2a). ET1, which showed a methylation pattern similar to that of normal cells, contained tumors of all subtypes. Epitypes ET2–ET5 were associated with luminal cancers and showed, from ET2 to ET5, a gradual decrease in the fraction of luminal A tumors and an increase in the fraction of luminal B tumors. In addition, the luminal epitypes showed a gradual shift away from the methylation pattern of normal cells (Fig. 1). ET6 showed an association with HER2-enriched tumors, and ET7 contained the majority of basal-like tumors.

The separation of luminal tumors into luminal A and B is based primarily on differential expression of proliferation-related genes. However, the expression of proliferation-related genes in luminal tumors is a continuum [37]. Hence, the separation into luminal A and B depends on the cutpoint in a continuous distribution. This cutpoint is typically highly dependent on the composition of tumors in the analyzed dataset, making robust assignment of individual tumors to luminal subtypes particularly difficult [37–39]. Therefore, we investigated the activity of eight breast cancer–specific gene expression modules [26] in both the discovery and validation cohorts and further substantiated the association between epitypes and gene expression phenotypes (Fig. 2b, Additional file 2: Figure S3). As expected, the steroid response module showed high activity in epitypes ET2–ET5 and low activity in ET7. The proliferation-related mitotic progression module displayed increasing expression levels across the luminal epitypes from ET2 (lowest levels) to ET5 (highest levels), in agreement with proliferative rates as measured by the fraction of cells in S phase determined by flow cytometry (Fig. 1c). Moreover, the basal module, containing basal cell keratins and known to display relatively high expression in normal breast tissue [1, 26], showed decreased expression levels across the luminal epitypes from ET2 (highest levels) to ET5 (lowest levels). Together, these results suggest that luminal tumors display promoter methylation patterns with a gradual shift away from normal cells associated with higher proliferative rate, lower normal cell content, and the luminal B subtype. Moreover, the results validate the strong association between DNA methylation patterns and gene expression phenotypes [6].

### Characteristics of CpG sites with breast cancer–specific methylation patterns

**Fig. 3:**
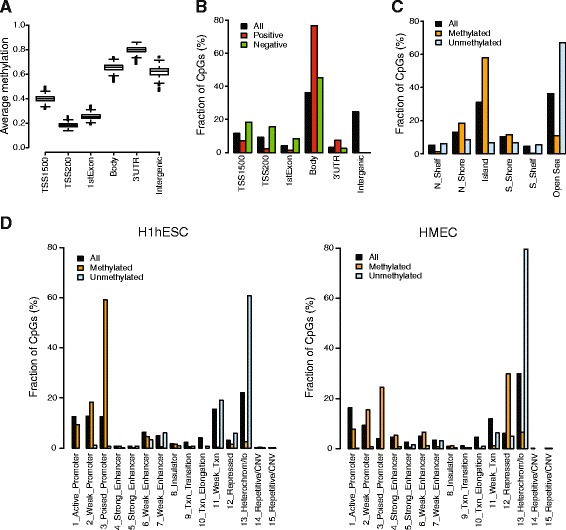
DNA methylation patterns in breast cancer. **a** Distribution of average β values for all CpG sites on the platform stratified by Illumina gene location across the 188 breast tumors in the discovery cohort. *TSS1500* = 1500 to 201 bp upstream of transcription start, *TSS200* = 200 bp to transcription start site, *Body* = gene body, *UTR* untranslated region. **b** A total of 324,991 CpG sites mapped to a unique gene in the validation gene expression data (All). Of these, 35,329 CpG sites showed a Pearson correlation greater than 0.2 (Positive) and 48,593 CpG sites showed a Pearson correlation less than −0.2 (Negative) between expression and methylation levels in the validation data. For a given set of CpG sites (All/Positive/Negative), a bar indicates the fraction of CpG sites assigned to the respective annotation. **c** Of all CpG sites on the platform (All), 1016 were identified as methylated in breast cancer (Methylated) and 1092 as unmethylated in breast cancer (Unmethylated). For a given set of CpG sites (All/Methylated/Unmethylated), a bar indicates the fraction of CpG sites assigned to the respective Illumina CpG island annotations. **d** For a given set of CpG sites (All/Methylated/Unmethylated), a bar indicates the fraction of CpG sites assigned to the respective chromatin state in human embryonic stem cells (H1hESC; *left*) and human mammary epithelial cells (HMEC; *right*). *CNV* copy number variation

Overall, DNA methylation patterns followed the expected pattern along gene structure, with low methylation levels near transcription start sites and high methylation levels in gene bodies, 3′ untranslated regions, and intergenic regions (Fig. 3a). Of the 324,991 CpG sites that mapped to a unique gene (a total of 18,797 genes) in the validation gene expression data, 35,329 CpG sites (7169 genes) showed a positive correlation (Pearson correlation greater than 0.2) and 48,593 CpG sites (10,724 genes) showed a negative correlation (Pearson correlation less than −0.2) between DNA methylation and gene expression levels in the validation cohort. A total of 4829 genes were associated with CpG sites with positive correlation as well as with CpG sites with negative correlation. CpG sites with negative correlation were enriched near transcription start sites, and CpG sites with positive correlation were enriched in gene bodies (Fig. 3b).

Of the 1016 CpG sites methylated in breast cancer compared with normal samples, 690 were annotated to a single gene corresponding to a total of 515 unique genes. Functional analysis of these hypermethylated genes using DAVID [27] showed significant enrichment for categories including homeobox genes (FDR = 2e-17), developmental proteins (FDR = 1e-15), and cell fate commitment (FDR = 8e-7). Of the 1092 CpG sites unmethylated in breast cancer, 645 were annotated to a single gene corresponding to a total of 416 unique genes. These hypomethylated genes showed significant enrichment for categories that included glycoproteins (FDR = 6e-15), keratinization (FDR = 3e-4), and epithelial cell differentiation (FDR = 0.008).

The CpG sites methylated in breast cancer compared with normal breast samples were enriched in CpG islands but also in shores, whereas the CpG sites unmethylated in breast cancer were enriched in open sea (Fig. 3c). Moreover, the CpG sites methylated in breast cancer overlapped with DNase I hypersensitive sites associated with open chromatin and active transcription as well as with regions bound by EZH2 in HMECs (Fig. 1). To investigate the genomic context of the CpG sites with tumor-specific methylation levels in more detail, we used 15 chromatin states based on genome-wide histone modification patterns and CTCF binding patterns in both H1hESCs and HMECs from the ENCODE Consortium [14, 29]. The chromatin states summarize coordinated chromatin marks. For example, trimethylation of lysine 27 on histone H3 (H3K27me3), H3K4me3, and dimethylation of lysine 4 on histone H3 (H3K4me2) jointly mark a bivalent, transcription-ready poised promoter state; H3K4me3, H3K4me2, histone H3 acetylated on lysine 27 (H3K27ac), and H3K9ac jointly mark an active promoter state; H3K27me3 alone marks a Polycomb-repressed state; and a heterochromatin/low signal state lacks histone marks [29]. CpG sites unmethylated in breast cancer compared with normal breast cells were located in genomic regions in the heterochromatin/low signal state in H1hESCs, and even more so in HMECs (Fig. 3d). CpG sites specifically methylated in breast cancer were located primarily in genomic regions in the poised promoter state in H1hESCs. Interestingly, in HMECs, the breast cancer methylated CpG sites were predominantly enriched in genomic regions in the poised promoter or in the Polycomb-repressed state (Fig. 3d). These results confirm the widespread observation that DNA methylation in cancer occurs in genes with promoters marked by Polycomb-mediated H3K27me3 in embryonic stem cells [40], but they also suggest a potential to further characterize methylation patterns in breast cancer by using histone modification patterns from mammary cells [6].

### Identification of CpG sites with epitype-specific hypermethylation

We observed a gradual increase in methylation across the luminal epitypes, ET2–ET5, of the CpG sites methylated in breast cancer, whereas only a subset of these CpG sites appeared to be methylated in the basal-like epitype ET7 (Fig. 1a). Consequently, we decided to investigate the characteristics of CpG sites methylated in basal-like breast cancer and specifically in luminal breast cancer, separately. We first identified 39 CpG sites among the 1016 breast cancer methylated CpG sites that were methylated in ET7 by selecting CpG sites having an average β value greater than 0.5 across the tumors in ET7 using the discovery cohort. These CpG sites were not specific to ET7 but were also methylated in ET5 (Additional file 2: Figure S4A). None of the breast cancer methylated CpG sites had an average β value greater than 0.5 in ET7 and an average β value less than 0.1 in ET5, indicating that methylation in the basal-like epitype ET7 is not epitype-specific, but rather reflects constitutive methylation present in both basal-like and some luminal breast cancers. Second, we identified 90 CpG sites that were specifically methylated in the luminal epitypes by selecting CpG sites having an average β value greater than 0.5 across the tumors in the hypermethylated luminal epitype ET5 and an average β value less than 0.1 across the tumors in ET7 using the discovery cohort. The CpG sites methylated in ET5 displayed a gradual increase in methylation across the luminal epitypes ET2–ET5 (Additional file 2: Figure S4B). The methylation patterns of the sets of CpG sites methylated in ET7 and ET5 were both validated in the validation cohort (Additional file 2: Figure S4).

### Functional characteristics of CpG sites with epitype-specific hypermethylation

Next, we investigated the characteristics of CpG sites methylated in ET7 and ET5, separately. Of the 39 CpG sites methylated in ET7, 20 were annotated to a single gene, corresponding to a total of 17 unique genes. Of the 90 CpG sites methylated in ET5, 74 were annotated to a single gene, corresponding to a total of 67 unique genes. Functional analysis of these two gene sets using DAVID did not identify significant enrichment (FDR < 0.01) for any categories except for zinc finger domains in the genes methylated in ET7 (FDR = 0.003). To gain further insight into the functions of genes in these two gene sets, we used gene set enrichment analysis to investigate their overlap with genes in 10,348 gene sets collected in the MSigDB [28]. The genes methylated in ET7 displayed significant overlap with gene sets containing Polycomb-repressed genes in human embryonic stem cells (FDR = 0.009 for H3K27me3 targets, FDR = 0.04 for SUZ12 targets, FDR = 0.04 for EED targets) [41]. The gene sets with most significant overlaps with genes methylated in ET5 fell into two categories. First, they were also enriched in gene sets of Polycomb-repressed genes in human embryonic stem cells (FDR = 2e-9 for H3K27me3 targets, FDR = 5e-8 for SUZ12 targets, FDR = 5e-8 for EED targets) [41]. Second, they were enriched in gene sets associated with different expression patterns between luminal and basal-like cells: genes downregulated in luminal-like cell lines compared with mesenchymal-like ones (FDR = 1e-9) [42], genes upregulated in the basal subtype (FDR = 3e-8), and genes downregulated in the luminal B subtype of breast cancer (FDR = 8e-7) [43], as well as genes upregulated in mammary stem cells (FDR = 3e-6) and genes downregulated in mature mammary luminal cells (FDR = 7e-6) in both mouse and human species [44]. These findings support our previous observation, based on a limited set of CpG sites, that many genes with subtype-specific expression may be regulated through methylation in breast cancer [6].

### Genomic characteristics of CpG sites with epitype-specific hypermethylation

**Fig. 4:**
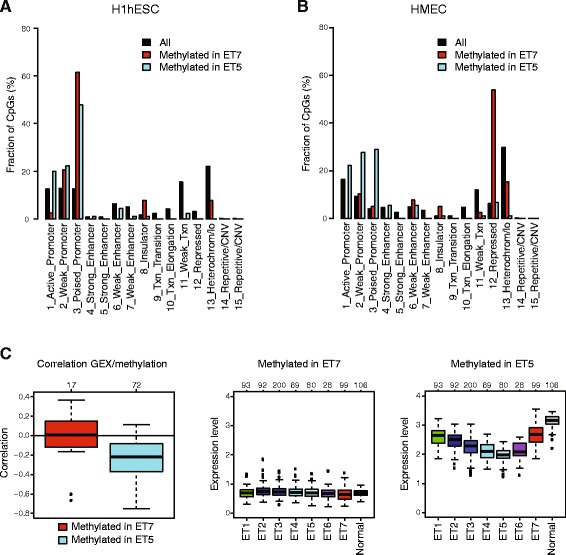
Characteristics of epitype-specific methylation patterns. Using the discovery cohort, 39 CpG sites were selected as methylated in the basal-like epitype ET7, and 90 CpG sites were selected as methylated in the luminal epitype ET5 but not in ET7. For a given set of CpG sites (All/Methylated in ET7/Methylated in ET5), a bar indicates the fraction of CpG sites assigned to the respective chromatin state in (**a**) human embryonic stem cells (H1hESC) and (**b**) human mammary epithelial cells (HMEC). **c** Pearson’s correlations between gene expression and methylation levels in the validation cohort for the CpG sites selected as methylated in ET7 and ET5 (*left*). The numbers on top indicate the number of CpG sites matched to a gene with gene expression data. The average gene expression levels across 661 breast tumors in the validation cohort stratified by epitype and 106 normal breast tissue samples from The Cancer Genome Atlas for the CpG sites methylated in ET7 (*center*) and methylated in ET5 (*right*) and matched to a gene with gene expression data. *GEX* gene expression, *CNV* copy number variation

The CpG sites methylated in ET5 were located primarily in islands (68 %) but also in shores (23 %), whereas the CpG sites methylated in ET7 were located in islands (44 %) and shores (49 %) in similar proportions. In H1hESCs, the sets of CpG sites methylated in ET7 and ET5 were both located primarily in genomic regions in promoter states, in particular in the poised promoter state (Fig. 4a). However, in HMECs, the CpG sites methylated in ET7 were not enriched in genomic regions in the same chromatin states as the CpG sites methylated in ET5 (Fig. 4b). In HMECs, the CpG sites methylated in ET5 were enriched in regions in weak promoter and poised promoter states, whereas the majority of the CpG sites methylated in ET7 were located in regions in the Polycomb-repressed state. We used validation cohort gene expression data to further substantiate the differences between these two methylation patterns. We matched 17 of the CpG sites methylated in ET7 to 16 unique genes and 72 of the CpG sites methylated in ET5 to 66 unique genes in the validation gene expression data. The CpG sites methylated in ET7 displayed no correlation between methylation and expression levels, and the genes were expressed at very low levels both in normal breast tissue and across breast cancers of all epitypes (Fig. 4c). On the contrary, the CpG sites methylated in ET5 displayed negative correlations between expression and methylation levels, and the genes were expressed in normal breast tissue and displayed decreasing expression levels across the luminal epitypes ET2–ET5 in concordance with their methylation levels (Fig. 4c, Additional file 2: Figure S4B). Taking these data together, by integrating genomic data from multiple levels, we show that DNA methylation in luminal and basal-like breast tumors is associated with different chromatin states and different gene expression patterns.

### Identification of CpG sites with epitype-specific hypomethylation

Most CpG sites unmethylated in breast cancer compared with normal samples were hypomethylated in the globally hypomethylated luminal epitype ET4 (Fig. 1a). However, hypomethylation in luminal tumors displayed two dominant patterns: Some CpG sites displayed hypomethylation more restricted to ET4, whereas others were hypomethylated in ET4 as well as in other luminal epitypes, in particular in ET5 (Fig. 1a). We also observed that there appeared to be a set of CpG sites specifically hypomethylated in the basal-like epitype ET7 (Fig. 1a). Consequently, we decided to investigate epitype-specific hypomethylation in breast cancer by identifying CpG sites belonging to these three patterns: hypomethylation specific to ET4, hypomethylation across luminal epitypes, and hypomethylation specific to ET7.

First, we identified 110 CpG sites among the 1092 CpG sites unmethylated in breast cancer that were specifically unmethylated in the globally hypomethylated epitype ET4 by selecting CpG sites having an average β value less than 0.5 across the tumors in ET4 and an average β value greater than 0.7 across the tumors in both ET5 and ET7 using the discovery cohort (Additional file 2: Figure S5A). Of these CpG sites, 48 were annotated to a single gene corresponding to 37 unique genes. Second, we identified 261 CpG sites that were unmethylated in luminal breast cancer by selecting CpG sites having an average β value less than 0.5 in ET5 and an average β value greater than 0.7 in ET7 using the discovery cohort. These CpG sites displayed gradual demethylation across the luminal epitypes from ET2 to ET5 (Additional file 2: Figure S5A), and 176 of them were annotated to a single gene corresponding to 156 unique genes. Third, we identified 15 CpG sites among the 1092 breast cancer unmethylated CpG sites that were specifically unmethylated in ET7 by selecting CpG sites having an average β value less than 0.5 across the tumors in ET7 and an average β value greater than 0.7 across the tumors in ET4 using the discovery cohort (Additional file 2: Figure S5A). Of the 15 CpG sites, 9 were annotated to a single gene corresponding to a total of 8 unique genes. The methylation patterns across epitypes of all three sets of hypomethylated CpG sites were validated in the TCGA validation cohort (Additional file 2: Figure S5A).

### Characteristics of CpG sites with epitype-specific hypomethylation

Both the set of CpG sites demethylated specifically in ET4 and the set demethylated gradually across luminal epitypes were located primarily in heterochromatin regions in HMECs (Additional file 2: Figure S5B) and displayed positive correlation between methylation and expression levels (Additional file 2: Figure S5C), but they were associated with genes with very low expression in breast cancer (Additional file 2: Figure S5D). These observations are consistent with findings of Hon et al. that regions undergoing hypomethylation in breast cancer typically are maintained in a transcriptionally silent state [13]. The CpG sites hypomethylated in luminal cancer were located primarily in open sea (77 %) and shores (10 %) but not in islands (0.4 %), whereas the CpG sites hypomethylated specifically in ET4 were located in open sea (47 %), islands (24 %), and shores (18 %). Hypomethylation of DNA in cancer has been shown to occur at long interspersed nuclear element (LINE) and long terminal repeat (LTR) repetitive elements, as well as at chromosome ends [45–48]. We found that the CpG sites demethylated in luminal cancer were significantly associated with LINE/LTR repetitive elements (*p* = 0.02 by Fisher’s exact test; *df* = 1) (Additional file 2: Figure S5F), whereas the CpG sites specifically demethylated in ET4 displayed enrichment within the first or last 5 Mb of chromosomes (Additional file 2: Figure S6G) and displayed shorter distances to the nearest chromosome end than the other CpG sites on the platform (*p* = 4e-21 by *t* test). It has been observed that DNA repeats can have a confounding effect on methylation measurements [49]. Our selection criteria identify CpG sites with larger methylation changes than the typical size of this erroneous effect [50]. Nevertheless, it is clear that use of bisulfite sequencing will help to reveal more details of hypomethylation of repeats in luminal cancers. We conclude that demethylation specific to ET4 and demethylation more common to all epitypes of luminal cancer may have different implications for genome function and progression of luminal tumors.

The CpG sites specifically unmethylated in ET7 were very few, were slightly enriched in enhancer regions in HMECs (Additional file 2: Figure S5B), and were primarily in shores (47 %) and in open sea (47 %). These CpG sites displayed limited correlation between expression and methylation levels but were associated with genes expressed in breast cancer using the validation cohort gene expression data (Additional file 2: Figure S5C, D). Because tumors of epitype ET7 displayed high expression levels of immune response genes (Additional file 2: Figure S3), we investigated the methylation levels of these CpG sites in a cohort of various subpopulations of blood cells [15]. We found that these CpG sites displayed, on average, decreased methylation levels in blood cells (Additional file 2: Figure S5E), as well as significant variation in methylation levels across subpopulations of blood cells, compared with all CpG sites on the arrays (*p* = 0.02 by *t* test). Hence, CpG sites specifically hypomethylated across basal-like breast cancer have limited influence on expression levels and may reflect low methylation levels in infiltrating subpopulations of normal cells, rather than de novo demethylation in tumor cells.

### Epitypes are associated with DNA copy number aberrations

**Fig. 5:**
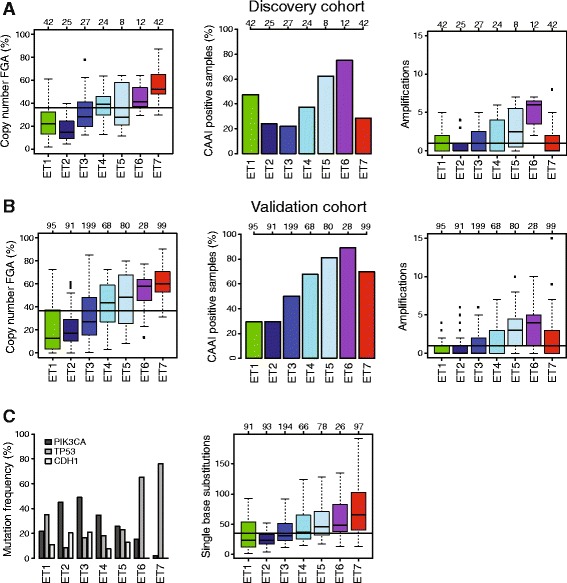
DNA copy number and somatic mutation characteristics of breast cancer epitypes. Amount of copy number alterations [fraction of the genome altered (FGA)], percentage of breast tumors classified as complex arm-wise aberration index–positive (CAAI positive), and the number of amplifications per tumor stratified by epitype in the (**a**) discovery cohort and (**b**) validation cohort. **c** The fraction of tumors in each epitype with a mutation in *PIK3CA*, *TP53*, and *CDH1* (*left*) and the total number of single base substitutions per tumor stratified by epitype (*right*) for 645 tumors in the validation cohort with exome sequence data. The number of samples is indicated on top for each epitype and cohort. *Horizontal lines* indicate median values across the cohort

We investigated DNA copy number changes across the epitypes in both the discovery and validation cohorts. Across the luminal epitypes from ET2 to ET5, we found that the FGA by copy number increased, that the alterations appeared increasingly more complex in terms of the CAAI [35], and that the number of amplifications per sample increased (Fig. 5a, b). Tumors of ET6, associated with HER2-enriched tumors, displayed the most complex copy number profiles, the largest numbers of amplifications per sample, and higher FGA than the luminal epitypes. Although tumors of the basal-like epitype ET7 displayed the largest FGA, the alterations were typically not as complex, and these tumors harbored few amplifications. Reassuringly, we note that the results were very similar for both the discovery and validation sets and conclude that the results were robust across both different tumor cohorts and copy number technologies (BAC arrays and Affymetrix Genome-Wide Human SNP Array 6.0 arrays).

With the exception of amplification of 17q12 (*ERBB2*), we did not find any particular associations between specific amplifications and epitypes (Additional file 2: Figure S6A). *ERBB2* was amplified in 83 % of ET6 tumors in the discovery cohort and in 46 % of ET6 tumors in the validation cohort. However, it should be noted that only a relatively small fraction of *ERBB2*-amplified cases are in ET6 [10 (21 %) of 48 in the discovery cohort and 13 (22 %) of 60 in the validation cohort]. Similar results were obtained using the gene expression–based subtype HER2-enriched (in the discovery cohort 7 (22 %) of 32 of HER2-enriched tumors in ET6 and 17 (23 %) of 73 in the validation cohort). A scheme to classify breast cancers into ten clusters based on CNAs that influence gene expression patterns (IntClust) has been proposed [51]. In agreement with our findings for individual amplifications, we observed only a moderate correspondence between IntClust groups and epitypes (Additional file 2: Figure S6B). In more detail, IntClust 10 is the dominant group in ET7, IntClust 5 is the dominant group in ET6, IntClust 3 is largest in ET2 and ET3, and ET4 and ET5 are very mixed with respect to IntClust groups. Taken together, our analyses of copy number changes demonstrate a strong association between epitypes and global patterns of genomic instability. Although specific aberrations in general were not associated with the epitypes, we identified a small subtype enriched for *ERBB2*-amplified tumors characterized by the most complex aberrations and a relatively large number of amplifications in addition to *ERBB2*.

### Epitypes are associated with mutations

We screened for associations between somatic mutations and epitypes using TCGA exome sequencing data for the validation set. There were 2205 genes with nonsilent mutations in at least 5 tumors in the validation set. Of these, only three genes, *PIK3CA*, *TP53* and *CDH1*, were significantly associated with the epitypes correcting for multiple testing [χ^2^ test (*df* = 6), FDR = 1 %] (Fig. 5c). Overall, the total number of mutations increased from ET2 to ET7 (Fig. 5c). Germline mutation data were available for only the discovery cohort. Patients with germline mutations in *BRCA2* were enriched in ET4 in the discovery cohort [8 (53 %) of 15; *p* = 8e-5, Fisher’s exact test (*df* = 1)]. In the discovery cohort, 22 (85 %) of 26 tumors from patients harboring *BRCA1* germline mutations were classified as ET7 [*p* = 1e-12, Fisher’s exact test (*df* = 1)]. In the validation cohort, 7 (58 %) of 12 tumors with somatic nonsilent *BRCA1* mutations were classified as ET7 [*p* = 6e-4, Fisher’s exact test (*df* = 1)].

### *BRCA1* and *HORMAD1* promoter methylation

Tumors of the basal-like epitype ET7 displayed highly altered genomes, but limited epitype-specific hyper- and hypomethylation, compared with normal samples. We investigated the promoter methylation status of *BRCA1* and *HORMAD1* to explore if aberrant methylation of these candidate drivers of genomic instability in basal-like breast cancer [52, 53] was associated with subsets of ET7 tumors. Using the 450K methylation data, we identified 11 (6 %) and 15 (2 %) *BRCA1* promoter methylated tumors in the discovery and validation cohorts, respectively. *BRCA1* promoter methylation status obtained using pyrosequencing was used to validate the 450K data. All 71 tumors in the discovery cohort analyzed using pyrosequencing displayed identical *BRCA1* promoter methylation status with both techniques. *BRCA1* promoter methylated tumors were primarily of the ET7 epitype [10 (91 %) of 11 in discovery cohort, 12 (80 %) of 15 in validation cohort]. The median correlation of methylation of the 21 promoter CpG sites used to assess *BRCA1* methylation status and *BRCA1* expression was −0.34 across the validation cohort. These CpG sites were all in the chromatin state active promoter in both H1hESCs and HMECs. Using the 450K methylation data, we identified 24 (13 %) and 47 (7 %) *HORMAD1* promoter unmethylated tumors in the discovery and validation cohorts, respectively. *HORMAD1* promoter unmethylated tumors were primarily of the ET7 epitype (23 (96 %) of 24 in discovery cohort, 40 (85 %) of 47 in validation cohort). The median correlation of methylation of the seven promoter CpG sites used to assess *HORMAD1* methylation status and *HORMAD1* expression was −0.63 across the validation cohort. These CpG sites were all in the heterochromatin/low signal chromatin state in both H1hESCs and HMECs. *BRCA1* methylation and *HORMAD1* demethylation were not mutually exclusive (6 of 11 BRCA1 methylated tumors were *HORMAD1* unmethylated in the discovery cohort and 3 of 15 in the validation cohort). These results demonstrate a strong association of *BRCA1* methylation and *HORMAD1* demethylation with the basal-like epitype ET7. Moreover, these candidate drivers of genomic instability in basal-like breast cancer may be regulated by aberrant methylation in subsets of these tumors.

### Methylation differences according to *BRCA1* status within the basal-like epitype

It is unclear if *BRCA1* deficiency is associated with a different epigenetic entity within basal-like tumors. Therefore, we screened for global methylation differences within the ET7 epitype between *BRCA1* promoter methylated tumors, *BRCA1* germline mutated tumors, and tumors with no known *BRCA1* aberration [*BRCA1* wild type (WT)]. We identified only 18 CpG sites with different methylation levels between the three tumor groups in the discovery cohort, even at relatively nonstringent statistical significance (Kruskal-Wallis test, FDR = 10 %), and all of these CpG sites were located around the *BRCA1* transcription start site. Similar results were obtained comparing *BRCA1* methylated tumors with only *BRCA1* WT tumors. In the validation cohort, we identified 90 CpG sites with different methylation levels between *BRCA1* WT, *BRCA1* methylated, and *BRCA1* somatically mutated tumors (Kruskal-Wallis test, FDR = 1 %). Of these, 29 CpG sites were located around the *BRCA1* transcription start site, while the other CpG sites were scattered across the genome. One sample with both a somatic missense mutation and promoter methylation were assigned to the methylation group in these analyses. Comparing *BRCA1* methylated tumors with only *BRCA1* WT tumors in the validation cohort, we identified 31 significant CpG sites (Wilcoxon test, FDR = 1 %); all but one located around the *BRCA1* transcription start site. Taken together, separating ET7 tumors into *BRCA1* promoter methylated, *BRCA1* mutated, or *BRCA1* WT revealed that these three groups have strikingly small differences in their genome-wide methylation patterns.

### Epitypes are associated with clinicopathological features

We investigated associations between epitypes and clinicopathological features for both the discovery and validation cohorts (Additional file 3: Tables S2 and S3, respectively). ER and PR status were significantly associated with epitypes in both the discovery cohort (*p* = 1e-17 and *p* = 7e-15, respectively; χ^2^ test) and the validation cohort (*p* = 1e-66 and *p* = 1e-54, respectively), as expected from the association between epitypes and gene expression phenotypes. Node status was more weakly associated with the epitypes with higher fractions of node positive tumors in ET4 and ET6 and lower fractions in ET7 (discovery cohort *p* = 0.02, validation cohort *p* = 0.03; χ^2^ test). Tumor size (in millimeters) was significantly associated with epitypes in the discovery cohort (*p* = 0.004; Kruskal-Wallis test), with the largest median tumor size in ET4–ET7 and the median tumor size increased across luminal epitypes from ET2 to ET4. Tumor size in millimeters was not available for the validation cohort. We therefore compared T1 tumors (≤20 mm) with larger tumors (T2 and T3) in this cohort and found that this dichotomized tumor size was associated with epitypes (*p* = 0.006; χ^2^ test). Our observations for the validation cohort were similar to the results in the discovery cohort: The largest fractions of large tumors (>20 mm) were in ET4–ET7, and the fraction of large tumors increased across the luminal epitypes from ET2 to ET5. In general, there were no associations between histological subtypes and epitypes. Lobular tumors were associated with the luminal epitypes (discovery cohort *p* = 0.11 and validation cohort *p* = 7e-7; χ^2^ test), but they were not associated with a particular luminal epitype. There was a significant association between age at diagnosis and epitype (discovery cohort *p* = 0.005 and validation cohort *p* = 5e-5; Kruskal-Wallis test). Convincingly, the oldest patients were in ET5 and the youngest patients in ET7 for both the discovery cohort (median age, cohort 48 years, ET5 66 years, ET7 45 years) and the validation cohort (median age, cohort 58 years, ET5 64 years, ET7 53 years).

**Fig. 6:**
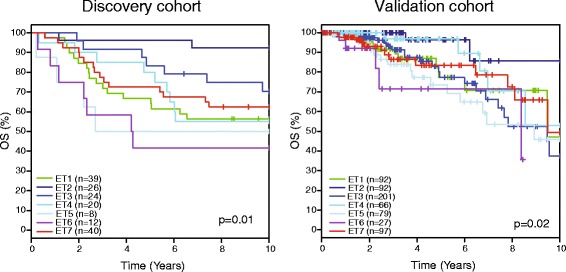
Association of breast cancer epitypes with patient outcome. Overall survival (OS) for breast cancer patients stratified by epitype in the discovery cohort (*left*) and the validation cohort (*right*). *p* Values were calculated using the log-rank test

The epitypes were associated with patient outcome in both the validation and discovery cohorts. The epitypes were associated with overall survival using both 10-year follow-up (discovery cohort *p* = 0.01, validation cohort *p* = 0.02; log-rank test) and the full follow-up (discovery cohort *p* = 0.01, validation cohort *p* = 0.04; log-rank test). Patient outcome analysis in the validation cohort is hampered by limited follow-up information. Reassuringly, the epitypes had similar overall survival patterns in both cohorts (Fig. 6). In particular, ET2 was associated with the best overall survival, ET5 and ET6 had the largest fractions of early events, and ET4 was characterized by a large fraction of events occurring 5 years after diagnosis.

## Discussion

DNA methylation of CpG sites in the genome is a normal developmental process that is of interest in cancer because many sites become aberrantly methylated or demethylated in the disease state. Moreover, it is often claimed that DNA methylation processes are of importance for tumor initiation and progression. We conducted a comprehensive analysis of genome-wide DNA methylation profiles of 188 breast tumor samples. Our overarching goal was to gain insights into how DNA methylation patterns on a genome-wide scale are associated with breast cancer heterogeneity. The findings were extensively validated in an independent cohort from TCGA encompassing 669 breast tumor samples. Previously, TCGA identified five epitypes—essentially corresponding to two luminal A epitypes, two luminal B epitypes, and a basal-like epitype—in an analysis of a large tumor set (*n* = 466) restricted to CpG sites in promoters [4]. The epitypes identified by TCGA provide a direct extension from the three epitypes typically identified in smaller studies [6–9]. In the present study, we identified seven epitypes of breast cancer using unsupervised analysis of genome-wide DNA methylation levels not restricted to CpG sites in promoters. Our epitypes give independent support to the five epitypes identified by TCGA and add a normal like epitype ET1 (normal-like tumors were very few in the original TCGA analysis) and an epitype ET6 enriched for HER2-enriched tumors. We performed an integrative analysis of genomic data at multiple levels to characterize the breast cancer epitypes.

To a large extent, the four luminal epitypes we identified (ET2–ET5) were characterized by a gradual increase of many variables from ET2 to ET5. For example, proliferative rate, fraction of luminal B tumors, promoter CpG island methylation levels, overall mutation rate, *TP53* mutation frequency, number and complexity of CNAs, number of amplifications, and tumor size all increased from ET2 to ET5. These findings are consistent with observations based on gene expression–based analyses suggesting that the separation of tumors into luminal A and luminal B is not well-defined, but rather reflects an arbitrary cutpoint in a continuous distribution of expression levels of proliferation-related genes [37–39]. Importantly, the luminal epitypes also displayed specific epigenetic characteristics in particular for the two more luminal B-like epitypes, ET4 and ET5. ET4 displayed a global hypomethylation phenotype and hypomethylation of subtelomeric regions, and was enriched for tumors with *BRCA2* germline mutations. However, the association between *BRCA2* germline mutations and ET4 remains to be validated in an independent dataset. ET5 displayed a global hypermethylation phenotype and was associated with older patients. The different global methylation patterns of ET4 and ET5 provide an example of the opportunities of going beyond analyses restricted to promoter CpG islands. On the contrary, the more luminal A-like epitypes ET2 and ET3 seemed to reflect more of a continuum, and the separation of these tumors into groups is likely cohort size–dependent. Indeed, in an unsupervised analysis of the large validation cohort (*n* = 669), there was support to separate ET3 into two groups (Additional file 2: Figure S2D).

HER2-enriched tumors are typically found to display heterogeneous DNA methylation patterns not associated with a specific epigenetic subtype [4, 6, 9]. In a previous study based on CpG sites in promoter regions, researchers identified a subtype associated with HER2-enriched tumors with a methylation pattern of infiltrating lymphocytes [54]. Such a subtype shows similarities to our epitype ET1 that contains relatively many HER2-enriched tumors and is characterized by high expression of immune response genes (Additional file 2: Figure S3). In the present study, we identified, for the first time to our knowledge, a breast cancer epitype associated with HER2-enriched tumors not displaying a methylation pattern similar to normal cells (ET6). ET6 contains only a fraction of the HER2-enriched or *ERBB2*-amplified tumors (around 20 %), and it is likely that our use of tumor sets containing many HER2-enriched tumors (Table 1) was essential to identifying this HER2-associated epitype. ET6 tumors were characterized by multiple amplifications beyond HER2 (the epitype with most amplifications per sample), the most complex genomes, *TP53* mutations, and poor overall survival.

We identified only a few associations between somatic mutations and epitypes in a screen taking multiple testing into account. As expected, *PIK3CA* and *CDH1* were frequently mutated in the luminal epitypes and *TP53* was frequently mutated in the basal-like and HER2-enriched epitypes. Many genes were mutated in relatively few samples, and it may be worthwhile to investigate whether mutations in sets of functionally related genes underlie specific epitypes. *BRCA1* mutations were significantly associated with the basal-like epitype (ET7). However, we did not identify any methylation differences within the basal-like epitype when stratified according to *BRCA1* status (either germline or somatic), with the exception that *BRCA1* alone displays promoter methylation in a subset of tumors with the basal-like epitype. These observations are consistent with findings reported by Prat et al., who observed very minor molecular differences at multiple levels (gene, protein, miRNA, and DNA methylation) according to *BRCA1* status in basal-like breast cancer [55].

Analyses of whole tumor tissues have revealed that DNA methylation patterns are heavily influenced by surrounding or infiltrating stromal cells [18, 54]. We identified an epitype with a methylation pattern similar to that of normal cells (ET1). By collecting tumors with normal-like methylation patterns into a separate epitype, the characteristics of the other epitypes are likely to become clearer. The reproducibility of identified subtypes is often assessed by showing that the proportion of cases assigned to each subtype is similar across different cohorts [51]. However, it is important to keep in mind that some methods have a bias toward keeping the proportions of subtypes similar [56]. In the present study, we analyzed retrospective tumor cohorts essentially generated by collecting as many tumors as possible, which may have resulted in cohorts with different characteristics. We found the proportions of samples assigned to the epitypes somewhat different for the discovery and validation cohorts. ET1 (23 % vs. 14 %), ET6 (6 % vs. 4 %), and ET7 (24 % vs. 15 %) contained larger fractions of samples in the discovery cohort, whereas ET3 (30 % vs. 15 %) and ET5 (12 % vs. 4 %) contained larger fractions of samples in the validation cohort. Reassuringly, these differences reflect differences in the composition of the cohorts. On one hand, the discovery cohort is enriched for HER2-enriched tumors [many of which likely are infiltrated by immune cells (Fig. 1a, Additional file 2: Figure S3)] and tumors from patients with *BRCA1* germline mutations. On the other hand, the validation cohort contains a larger fraction of ER-positive luminal tumors and more tumors from older patients (Table 1). These interpretable connections between cohort composition and epitype proportions add support to the reproducibility and generalizability of our epitypes.

Traditionally, epigenetic reprogramming has been thought to contribute to tumor progression by silencing tumor suppressor genes. This model has been challenged by the finding that most cancer-associated methylation occurs in genes that are already repressed in the normal tissue from which the cancer derives [57, 58]. We identified two different patterns of cancer-associated DNA methylation in breast tumors. One set of CpG sites was methylated in both luminal and basal-like breast tumors and was thereby considered constitutive, whereas a second set was specifically methylated in luminal breast cancer. We found that the set of CpG sites with constitutive methylation matched the paradigm of being repressed in normal breast epithelial cells and displaying no correlation between expression and methylation levels. On the contrary, the set of CpG sites methylated specifically in luminal breast cancer were associated with genes expressed in normal breast epithelial cells and displayed negative correlation between expression and methylation levels. Similar observations have been made in pediatric acute lymphoblastic leukemia for CpG sites with constitutive and subtype-specific methylation patterns, respectively [59]. Moreover, differentially methylated regions associated with bladder cancer subtypes have been found to separate into patterns with substantial differences with respect to expression–methylation correlations [48]. As proposed by Sproul et al., the aberrant constitutive methylation in breast tumors may be a marker of their epithelial cell lineage rather than of tumor progression [60]. However, the CpG sites specifically methylated in luminal breast cancer do influence gene expression levels and may contribute to tumor progression. Methylation of these CpG sites was associated with epitypes enriched for luminal B tumors. This finding is consistent with our previously proposed model in which luminal differentiation is partially blocked by aberrant methylation in luminal B tumors [6].

Constitutive methylation in breast cancer and methylation specific to luminal cancer occurred in regions in different chromatin contexts in normal mammary epithelial cells. Constitutive methylation occurred primarily in regions in a Polycomb-repressed state, consistent with this methylation not being the original cause of repression of gene expression. Luminal-specific methylation was enriched in regions in active promoter states in normal cells, adding support to the picture in which aberrant methylation contributes to a block to keep some luminal cancers more undifferentiated. Because breast cancer–specific methylation to a large extent is associated with chromatin states and thus with aberrant methylation of very many genes, often already repressed in precancerous tissue, it is not straightforward to identify potential epigenetic driver genes. It may be that epigenetically deregulated driver genes are rare and that most methylation in cancer is a passenger event of general epigenetic deregulation in cancer. Perhaps the methylated genes are prone to methylation merely because they are repressed in a tissue-specific fashion [58]. Moreover, we observed that genes unmethylated in breast cancer were associated with subtelomeric regions and DNA repeats and showed limited influence on gene expression levels. Hence, identification of candidate tumor suppressor genes or oncogenes based solely on methylation data will likely result in numerous false-positive findings.

We focused our analyses on genome-wide screens for CpG sites that display changes in methylation state between macrodissected tumor tissue and normal breast tissue. There are limitations with use of this approach, although its utility in identifying and characterizing robust epitypes is clear. Directions for future improved characterization of breast cancer epigenetic heterogeneity include using different normal cell subpopulations separately instead of normal breast tissue, and investigating CpG sites that display varying or intermediate methylation in normal cell populations. Another limitation of the present study is that we restricted our analyses to epitype-specific methylation patterns. These analyses revealed very low numbers of CpG sites with specific hyper- and hypomethylation across the basal-like epitype. However, directed analyses showed that *BRCA1* and *HORMAD1* are clear candidates for driver genes directly regulated by aberrant methylation in some basal-like breast cancers. Taken together, our results suggest that the dominant patterns of breast cancer–specific hyper- and hypomethylation are associated with their genomic contexts, but also that there may be epigenetically deregulated driver genes for subsets of samples.

The gene expression–based molecular subtypes of breast cancer have been included in international guidelines for breast cancer treatment [61]. The epitypes of breast cancer described in this report reflect, to a large extent, the gene expression–based subtypes, and perhaps may not add independent prognostic value. Nevertheless, it could still be that DNA methylation measurement provides a technically simpler and more robust clinical subtyping tool. Systemic treatment decisions for luminal breast cancer are partly dependent on differences in proliferative rates used to separate these tumors into luminal A and B. Our characterization of luminal epitypes opens up new opportunities to evaluate connections between chemotherapy response and molecular characteristics of luminal tumors. For example, the identification of a luminal group of patients with very few relapses who could be spared chemotherapy may potentially be improved by integrating methylation data with other molecular information. Because aberrant methylation in breast cancer affects large numbers of CpG sites, there are likely very many individual CpG sites that correlate with prognostic information. It has been found that most genes methylated in breast cancer cell lines cannot be derepressed by using the demethylating agent 5-aza-2′-deocycytidine [60]. However, genes already repressed in normal epithelial cells dominated the evaluated genes. Hence, it may still be worthwhile to evaluate if demethylating agents have an effect on the subset of genes in luminal tumors with expression levels clearly associated with promoter methylation. Potentially, demethylating agents could result in further differentiation of luminal tumors with extensive promoter methylation and could benefit patient outcomes.

## Conclusions

We performed a comprehensive analysis of DNA methylation patterns in human breast tumors. The methylation epitypes we describe exemplify how integrating different types of molecular genome-wide data can improve the characterization of breast cancer heterogeneity. We identified differences in the methylation patterns across breast cancer subtypes. Although we showed that candidate driver genes such as *BRCA1* and *HORMAD1* display aberrant methylation in subsets of basal-like tumors, the results of our study imply that the dominant methylation patterns across basal-like breast cancer are passenger events reflecting the tissue of origin and infiltrating cells. However, methylation patterns specific to luminal breast cancer drive gene expression and may contribute to tumor progression in this subtype.

## Additional files

Additional file 1: Table S1. The 2108 CpG set with epitype centroids. (XLSX 357 kb) Additional file 2: Figures S1–S6. All supplementary figures with legends. (PDF 2964 kb) Additional file 3: Tables S2 and S3. Clinicopathological features for the seven epitypes in the discovery and validation cohorts. (PDF 71 kb)

## Abbreviations

BAC: bacterial artificial chromosome
CAAI: complex arm-wise aberration index
CNA: copy number aberration
CNV: copy number variation ER, estrogen receptor
ET: epitype cluster
FDR: false discovery rate
FGA: fraction of the genome altered
GEO: Gene Expression Omnibus
HER2: human epidermal growth factor receptor 2
H1hESC: human embryonic stem cell
H3K27ac: histone H3 acetylated on lysine 27
H3K4me2: dimethylation of lysine 4 on histone H3
H3K27me3: trimethylation of lysine 27 on histone H3
HMEC: human mammary epithelial cell
HMEndoC: human mammary endothelial cell
HMF: human mammary fibroblast
LINE: long interspersed nuclear element
LTR: long terminal repeat
MAF: mutation annotation format
MSigDB: Molecular Signatures Database
NCBI: National Center for Biotechnology Information
PR: progesterone receptor
TCGA: The Cancer Genome Atlas
TPM: transcripts per million
UTR: untranslated region
WT: wild type
