# Supplementary material for: An integrated genomics analysis of epigenetic subtypes in human breast tumors links DNA methylation patterns to chromatin states in normal mammary cells
Source: Breast Cancer Res. 2016 Feb 29;18:27. doi: 10.1186/s13058-016-0685-5 (PMC4770527; doi:10.1186/s13058-016-0685-5)
Supplement: Additional file 2: Figures S1–S6. — All supplementary figures with legends. (PDF 2964 kb) [file 13058_2016_685_MOESM2_ESM.pdf]

Figure S1. Identification of breast cancer epitypes in the discovery cohort. (A) Bootstrap clustering of 188 breast tumors using 2,108 CpGs and a seven-group cluster solution. The heatmap displays correlations between pairs of tumors. Row and column annotation bars represent the seven groups (epitypes). **(B)** Plot tracking group assignments of individual samples from bootstrap clustering of 188 breast tumors using 2,108 CpGs and solutions with three to seven sample clusters. Each column corresponds to a tumor sample. **(C)** Number of CpGs with differential methylation levels between 188 breast tumor samples and 96 normal samples as a function of number of tumors with differential methylation levels. 2,108 CpGs were identified as having differential methylation levels in at least 10 tumors as compared to normal tissue samples. **(D)** The seven bootstrap clusters (epitypes) are robust across a wide range of different CpG sets. The heatmap shows the overlap between boostrap analyses performed using different number of CpGs from (C) and a seven-group cluster solution. **(E)** Principal component analysis of technical factors, clinicopathological factors, and DNA methylation epitypes. The full set of CpGs with no missing values was used (n=356,331). The heatmap shows the significance of the association between a principal component (columns) and a specific factor (rows) [21]. In the heatmap a more intense color means that more variation is explained by a specific factor. **(F)** Distribution of tumors across three bisulphite conversion plates used in the sample labeling across the seven breast cancer epitypes. **(G)** Distribution of tumors from different BeadChips across the seven breast cancer epitypes.

Figure S2. Reproducibility of breast cancer epitypes in the TCGA validation cohort. (A) Global hypermethylation scores for all CpGs (left) and all CpGs in promoters and CpG islands (right) across the epitypes for the validation cohort. The number of samples in each epitype is indicated at the top. **(B)** Principal component analysis of technical factors, clinicopathological factors, and DNA methylation epitypes for the 669 tumors in the validation cohort. The full set of

CpGs with no missing values was used (n=454,553). The heatmap shows the significance of the association between a principal component (columns) and a specific factor (rows) [21]. In the heatmap a more intense color means that more variation is explained by a specific factor. Epitype corresponds to the classification of the validation cohort using the classifier for the seven epitypes constructed using the discovery cohort. **(C)** Distribution of tumors in different TCGA sample batches across the seven breast cancer epitypes. The total number of tumors per epitype is indicated at the top. **(D)** Distribution of tumors from different TCGA BeadChips across the seven breast cancer epitypes. **(E)** Overlap of epitype classification (based on the seven epitypes identified using 2,108 CpGs and the discovery cohort) of tumors in the TCGA validation cohort (vertical axis) with unsupervised bootstrap tumor groups identified in the TCGA validation cohort (horizontal axis) using an eight group solution and the same 2,108 CpGs. Matrix cells indicate the percentage of tumors in an unsupervised bootstrap group found in an epitype. The number of samples in each unsupervised bootstrap group is indicated at the top.

Figure S3. Activity of breast cancer specific gene expression modules across the epitypes. (A) Expression levels of eight breast cancer specific gene modules in the discovery cohort (the microarray data in the discovery cohort contained expression levels for 12 of 21 genes in the mitotic checkpoint module, 13 of 22 in the mitotic progression module, 1 of 8 in the basal module, 7 of 9 in the steroid response module 39 of 71 in the immune response module, 20 of 32 in the stroma module, 4 of 18 in the lipid module, and 5 of 6 in the early response module). **(B)** Expression levels of eight breast cancer specific gene modules in the validation cohort (The TCGA RNAseq data in the validation cohort contained expression levels for all genes in the modules, except for two genes in the immune response module).

Figure S4. Epitype-specific hypermethylation patterns. (A) The average methylation level of the CpGs methylated in ET7 in each tumor in the discovery cohort (left) and validation cohort (right) stratified by epitype. **(B)** The average methylation level of the CpGs methylated in ET5 in each tumor in the discovery cohort (left) and validation cohort (right) stratified by epitype. The number of tumors in each epitype is indicated at the top of each of the plots.

Figure S5. Epitype-specific hypomethylation patterns. (A) The average methylation level of the CpGs demethylated in ET4, demethylated in luminal tumors and demethylated in ET7 in each tumor in the discovery cohort (top) and validation cohort (bottom) stratified by epitype. The number of tumors in each epitype is indicated at the top of each of the plots. **(B)** For a given set of CpGs (All/Demethylated in ET4/Demethylated in luminal/Demethylated in ET7) a bar indicates the fraction of CpGs assigned to the respective chromatin state in HMEC. **(C)** Pearson correlation between gene expression and methylation levels for the CpGs selected as demethylated in ET7, luminal and ET4. The numbers on top indicate the number of CpGs matched to a gene with gene expression data. **(D)** The average gene expression levels across 661 breast tumors in the validation cohort stratified by epitype and 106 normal breast tissue samples from TCGA for the CpGs demethylated in ET4, luminal and ET7 and matched to a gene with gene expression data. **(E)** The average methylation levels across different subpopulations of blood cells for the CpGs demethylated in ET4, luminal and ET7. **(F)** For a given set of CpGs (All/Demethylated in ET4/Demethylated in luminal) a bar indicates the fraction of CpGs situated in different types of DNA repeats. **(G)** For a given set of CpGs (All/Demethylated in ET4/Demethylated in luminal) a bar indicates the fraction of CpGs with more or less than 5Mb to the nearest chrosmome end.

Figure S6. Amplifications and IntClust groups across epitypes. (A) The frequency of samples in

each epitype harboring specific amplifications for the discovery cohort (left) and the validation cohort (right). **(B)** The number of samples in each epitype classified into the ten IntClust groups for the discovery cohort (left) and the validation cohort (right). The number of tumors in each epitype is indicated at the top of each of the plots.

Figure S1

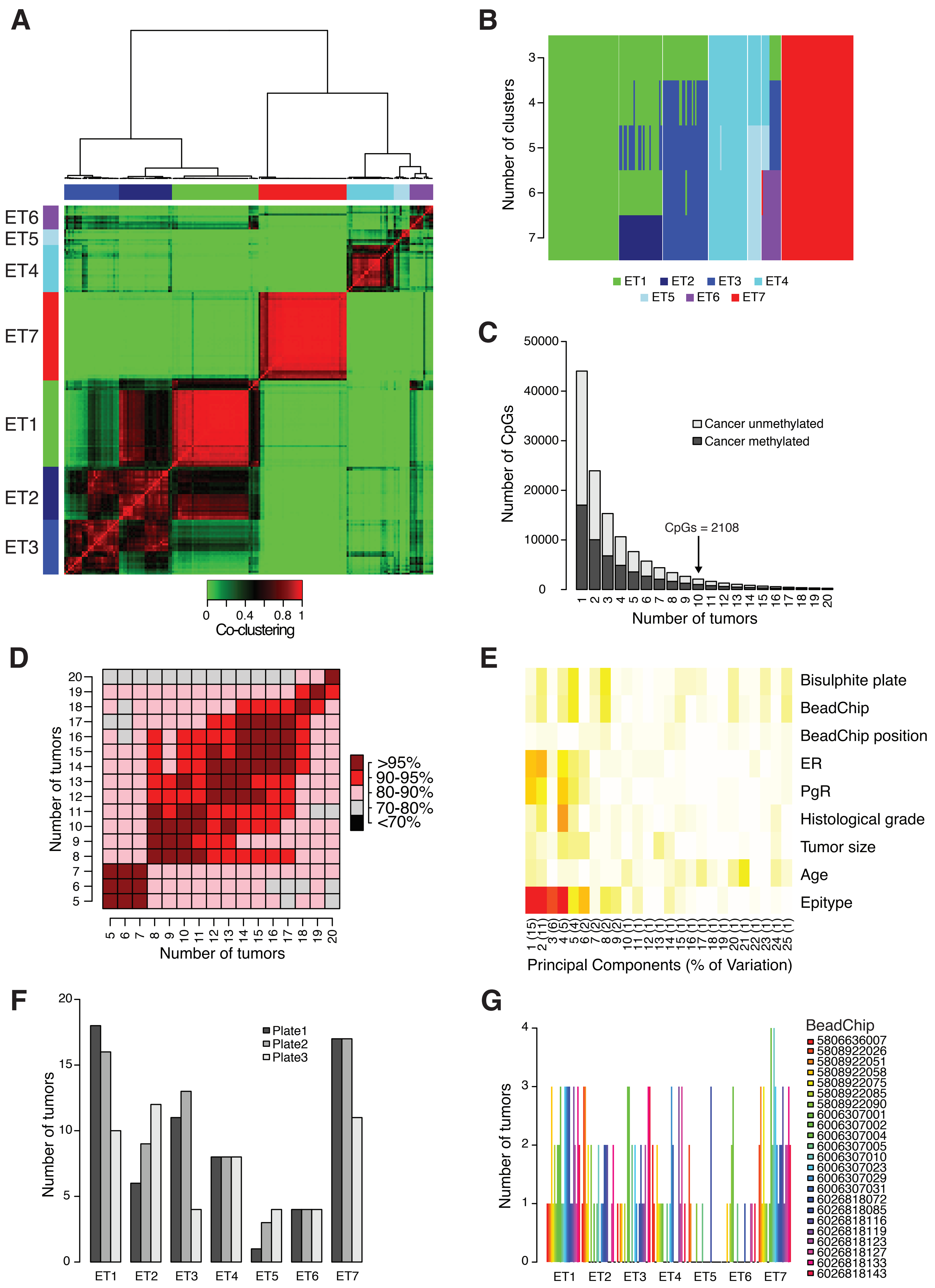

A
ET6
ET5
ET4
ET7
ET1
ET2
ET3
0
0.4
0.8
1
Co-clustering
B
Number of clusters
3
4
5
6
7
ET1
ET2
ET3
ET4
ET5
ET6
ET7
C
Number of CpGs
50000
40000
30000
20000
10000
0
Cancer unmethylated
Cancer methylated
CpGs = 2108
Number of tumors
D
Number of tumors
>95%
90-95%
80-90%
70-80%
<70%
Number of tumors
E
Bisulphite plate
BeadChip
BeadChip position
ER
PgR
Histological grade
Tumor size
Age
Epitype
1 (15)
2 (11)
3 (6)
4 (5)
5 (4)
6 (2)
7 (2)
8 (2)
9 (2)
10 (1)
11 (1)
12 (1)
13 (1)
14 (1)
15 (1)
16 (1)
17 (1)
18 (1)
19 (1)
20 (1)
21 (1)
22 (1)
23 (1)
24 (1)
25 (1)
Principal Components (% of Variation)
F
Number of tumors
Plate1
Plate2
Plate3
ET1
ET2
ET3
ET4
ET5
ET6
ET7
G
Number of tumors
BeadChip
5806636007
5808922026
5808922051
5808922058
5808922075
5808922085
5808922090
6006307001
6006307002
6006307004
6006307005
6006307010
6006307023
6006307029
6006307031
6026818072
6026818085
6026818116
6026818119
6026818123
6026818127
6026818133
6026818143
ET1
ET2
ET3
ET4
ET5
ET6
ET7

Figure S2

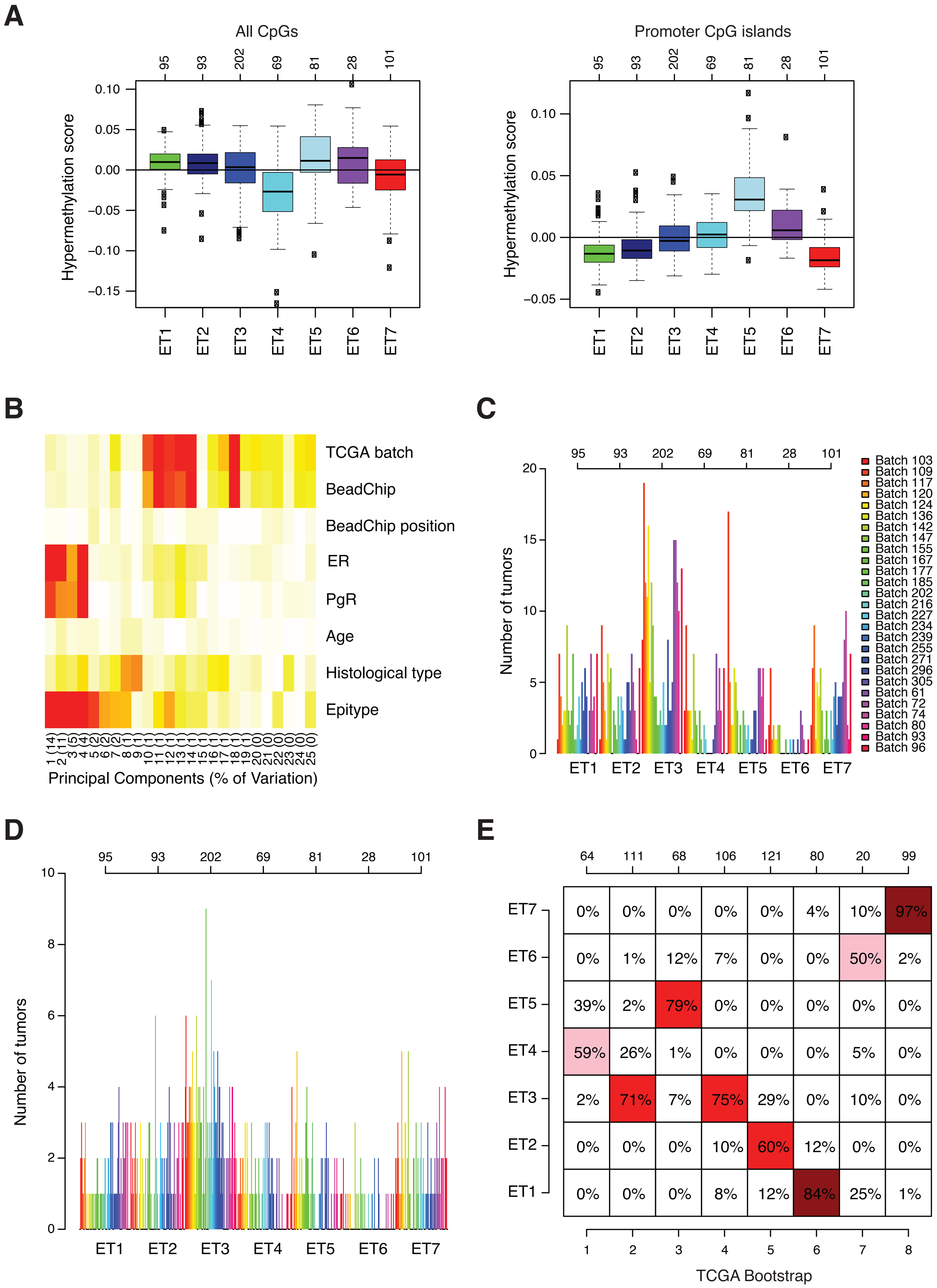
A
All CpGs
Promoter CpG islands
95
93
202
69
81
28
101
Hypermethylation score
0.10
0.05
0.00
−0.05
−0.10
−0.15
ET1
ET2
ET3
ET4
ET5
ET6
ET7
B
TCGA batch
BeadChip
BeadChip position
ER
PgR
Age
Histological type
Epitype
1 (14)
2 (11)
3 (5)
4 (4)
5 (2)
6 (2)
7 (2)
8 (1)
9 (1)
10 (1)
11 (1)
12 (1)
13 (1)
14 (1)
15 (1)
16 (1)
17 (1)
18 (1)
19 (1)
20 (0)
21 (0)
22 (0)
23 (0)
24 (0)
25 (0)
Principal Components (% of Variation)
C
Number of tumors
Batch 103
Batch 109
Batch 117
Batch 120
Batch 124
Batch 136
Batch 142
Batch 147
Batch 155
Batch 167
Batch 177
Batch 185
Batch 202
Batch 216
Batch 227
Batch 234
Batch 239
Batch 255
Batch 271
Batch 296
Batch 305
Batch 61
Batch 72
Batch 74
Batch 80
Batch 93
Batch 96
D
E
64
111
68
106
121
80
20
99
0% 0% 0% 0% 0% 4% 10% 97%
0% 1% 12% 7% 0% 0% 50% 2%
39% 2% 79% 0% 0% 0% 0% 0%
59% 26% 1% 0% 0% 0% 5% 0%
2% 71% 7% 75% 29% 0% 10% 0%
0% 0% 0% 10% 60% 12% 0% 0%
0% 0% 0% 8% 12% 84% 25% 1%
TCGA Bootstrap

Figure S3

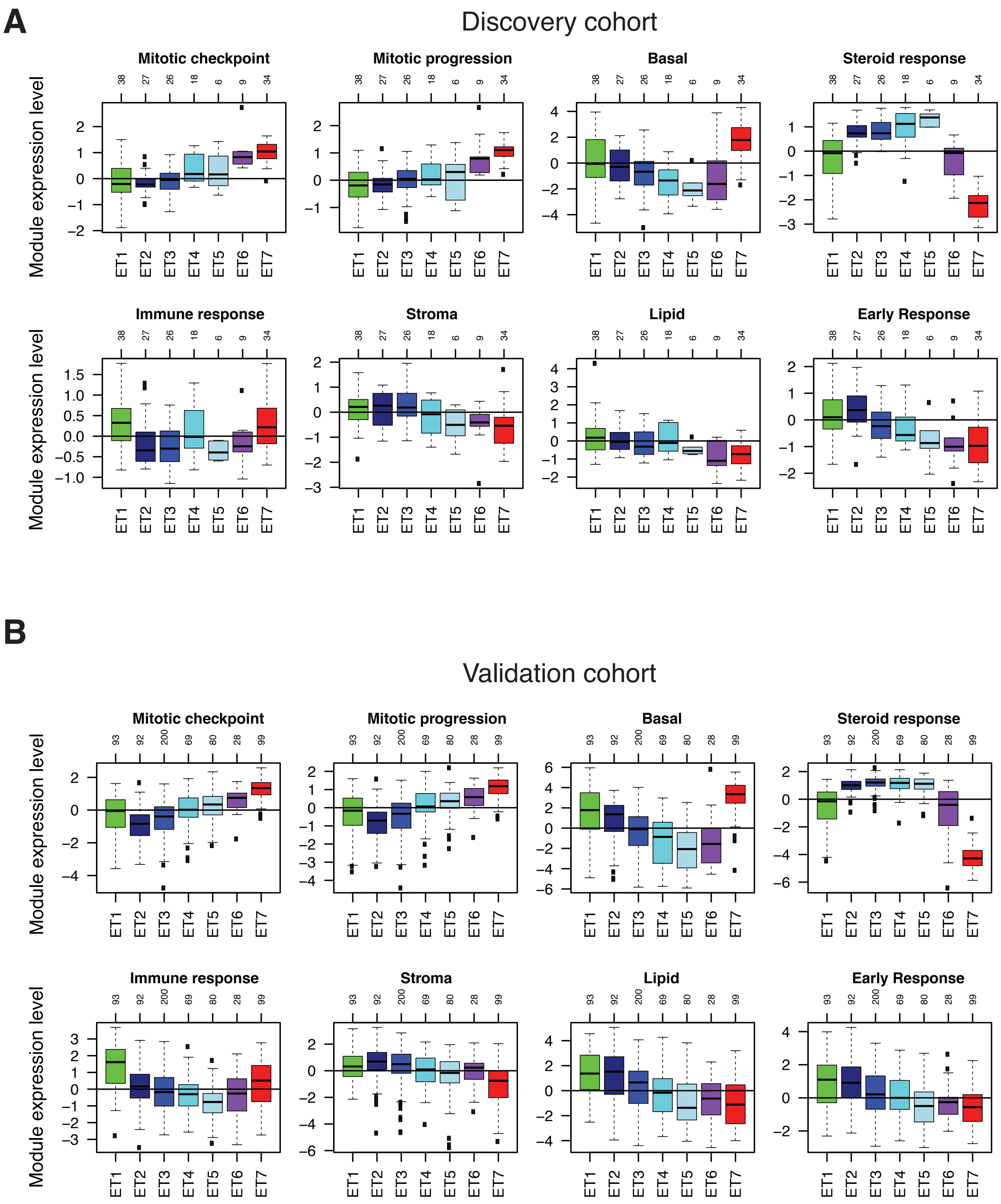

A
Discovery cohort
Mitotic checkpoint
Mitotic progression
Basal
Steroid response
Immune response
Stroma
Lipid
Early Response
Module expression level
38 27 26 18 6 9 34
ET1 ET2 ET3 ET4 ET5 ET6 ET7
B
Validation cohort
Mitotic checkpoint
Mitotic progression
Basal
Steroid response
Immune response
Stroma
Lipid
Early Response
Module expression level
93 92 200 69 80 28 99
ET1 ET2 ET3 ET4 ET5 ET6 ET7

Figure S4

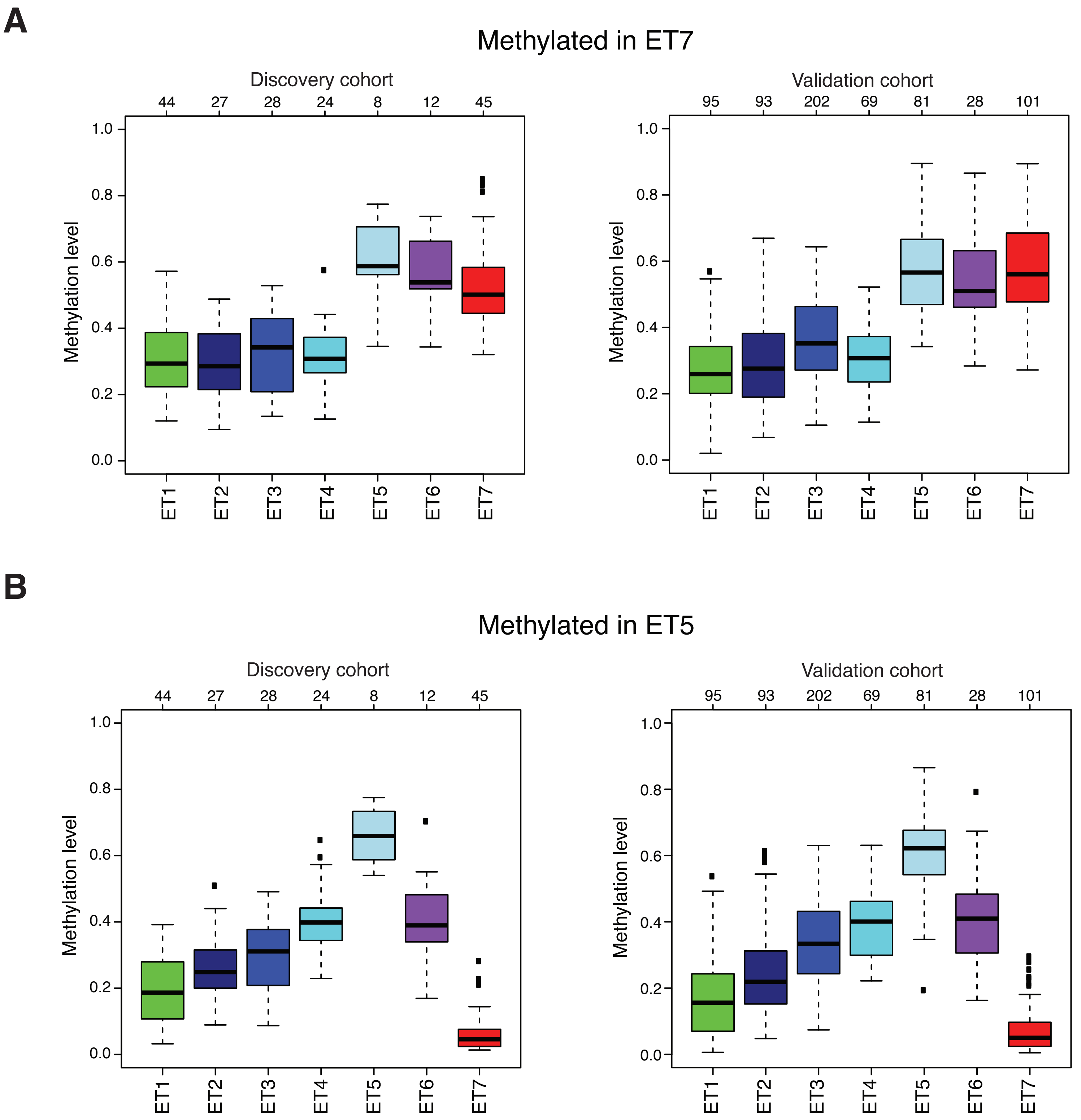

A
Methylated in ET7
Discovery cohort
44 27 28 24 8 12 45
Validation cohort
95 93 202 69 81 28 101
Methylation level
1.0
0.8
0.6
0.4
0.2
0.0
ET1 ET2 ET3 ET4 ET5 ET6 ET7
B
Methylated in ET5
Discovery cohort
44 27 28 24 8 12 45
Validation cohort
95 93 202 69 81 28 101
Methylation level
1.0
0.8
0.6
0.4
0.2
0.0
ET1 ET2 ET3 ET4 ET5 ET6 ET7

Figure S5

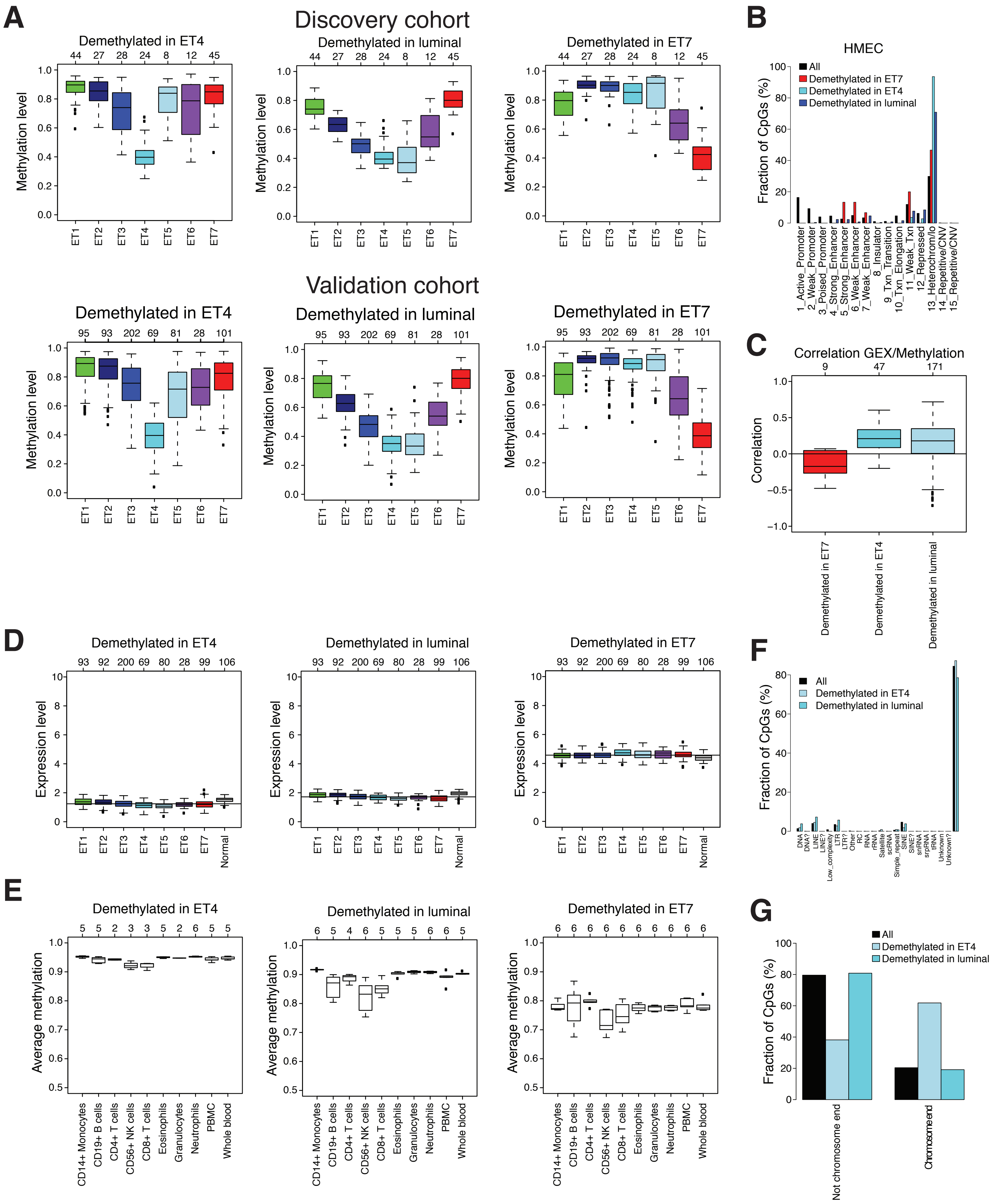
A
Discovery cohort
Demethylated in ET4
Demethylated in luminal
Demethylated in ET7
Methylation level
Validation cohort
B
HMEC
All
Demethylated in ET7
Demethylated in ET4
Demethylated in luminal
Fraction of CpGs (%)
1_Active_Promoter
2_Weak_Promoter
3_Poised_Promoter
4_Strong_Enhancer
5_Strong_Enhancer
6_Weak_Enhancer
7_Weak_Enhancer
8_Insulator
9_Txn_Transition
10_Txn_Elongation
11_Weak_Txn
12_Repressed
13_Heterochrom/lo
14_Repetitive/CNV
15_Repetitive/CNV
C
Correlation GEX/Methylation
Correlation
D
Expression level
Normal
E
Average methylation
CD14+ Monocytes
CD19+ B cells
CD4+ T cells
CD56+ NK cells
CD8+ T cells
Eosinophils
Granulocytes
Neutrophils
PBMC
Whole blood
F
G
Not chromosome end
Chromosome end

Figure S6

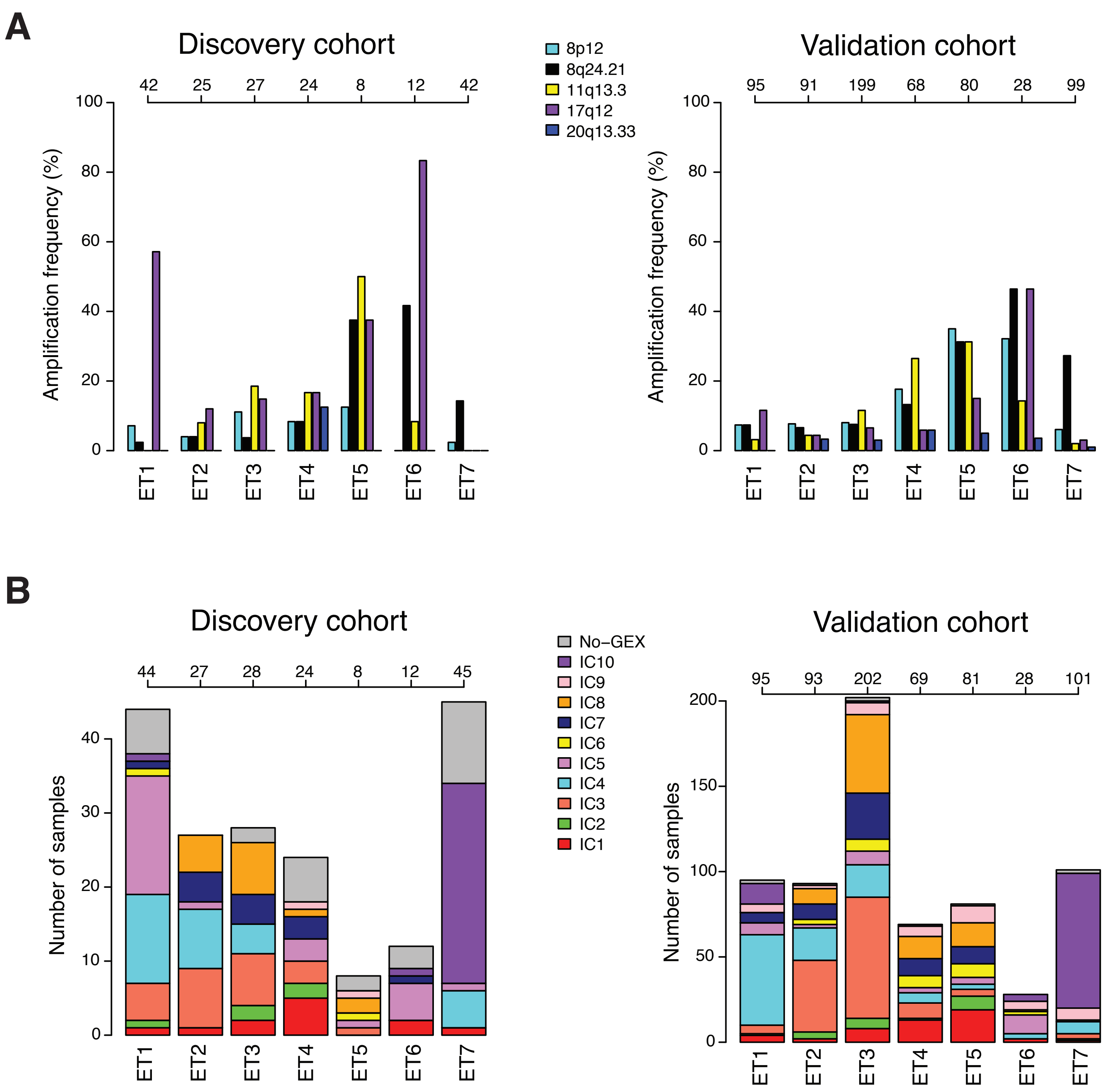
A
Discovery cohort
Validation cohort
Amplification frequency (%)
8p12
8q24.21
11q13.3
17q12
20q13.33
42 25 27 24 8 12 42
95 91 199 68 80 28 99
ET1 ET2 ET3 ET4 ET5 ET6 ET7
B
Discovery cohort
Validation cohort
Number of samples
No−GEX
IC10
IC9
IC8
IC7
IC6
IC5
IC4
IC3
IC2
IC1
44 27 28 24 8 12 45
95 93 202 69 81 28 101
ET1 ET2 ET3 ET4 ET5 ET6 ET7
