# Supplementary material for: An integrated genomics analysis of epigenetic subtypes in human breast tumors links DNA methylation patterns to chromatin states in normal mammary cells
Source: Breast Cancer Res. 2016 Feb 29;18:27. doi: 10.1186/s13058-016-0685-5 (PMC4770527; doi:10.1186/s13058-016-0685-5)
Supplement: Additional file 3: Tables S2 and S3. — Clinicopathological features for the seven epitypes in the discovery and validation cohorts. (PDF 71 kb) [file 13058_2016_685_MOESM3_ESM.pdf]

Table S2. Clinicopathological features for the seven epitypes in the discovery cohort

	ET1	ET2	ET3	ET4	ET5	ET6	ET7	P
**Group size**	44	27	28	24	8	12	45	
**ER status**								1.122217e-17
ER positive	16	25	23	21	7	4	0	
ER negative	23	2	3	3	1	7	38	
**PR status**								6.795e-15
PR positive	13	23	25	16	6	4	1	
PR negative	25	4	1	7	2	7	36	
**Node status**								0.01836
Node positive	19	6	9	8	2	7	8	
Node negative	16	21	14	7	5	3	25	
**Median tumor size (mm)**	20	15	19	24.5	25	25	25	0.003873
**Histological subtypes**								0.1996
Ductal	27	17	20	14	6	7	29	
Lobular	1	3	1	3	0	0	0	
Medullary	0	1	0	0	0	0	2	
Other	10	6	5	1	0	2	3	
**Median age at diagnosis**	46.64	50.64	47.56	49.65	66.28	57.28	44.73	0.004641

P values were calculated using Chi-square test for ER status, PR status, node status, and histological subtypes, and Kruskal-Wallis test was used for tumor size and age at diagnosis.

Table S3. Clinicopathological features for the seven epitypes in the validation cohort

	ET1	ET2	ET3	ET4	ET5	ET6	ET7	P
**Group size**	95	93	202	69	81	28	101	
**ER status**								1.149098e-66
ER positive	55	81	185	61	67	13	12	
ER negative	30	5	5	2	5	11	84	
**PR status**								1.397615e-54
PR positive	46	75	171	58	51	10	4	
PR negative	38	11	18	5	21	14	91	
**Node status**								0.03273593
Node positive	45	53	114	45	42	16	41	
Node negative	47	38	84	21	34	10	56	
**Tumor size**								0.005721
T1	32	36	56	13	12	6	21	
T2 or T3	59	53	140	52	63	17	73	
**Histological subtypes**								5.614e-06
Ductal	59	53	125	50	57	20	82	
Lobular	25	25	56	9	16	2	1	
Medullary	2	0	0	0	0	0	3	
Other	6	14	20	7	6	5	10	
**Median age at diagnosis**	54.5	58.0	57.0	57.5	64.0	58.0	53.0	5.426e-05

P values were calculated using Chi-square test for ER status, PR status, node status, tumor size and histological subtypes, and Kruskal-Wallis test was used for age at diagnosis.
